# Perinatal physiology and welfare in water buffalo: calving, mother–calf bonding, lactation, and postpartum disorders

**DOI:** 10.3389/fvets.2026.1913756

**Published:** 2026-08-24

**Authors:** Daniel Mota-Rojas, Fabio Napolitano, Vivian Fischer, Arthur Fernandes Bettencourt, Adolfo Álvarez-Macías, Ismael Hernández-Avalos, Dina Villanueva-García, Rosy Cruz-Monterrosa, J. Efrén Ramírez-Bribiesca, Adriana Domínguez-Oliva, Adriana Olmos-Hernández, Brenda Reyes-Sotelo, Fabiola Torres-Bernal, Antonio H. H. Minervino

**Affiliations:** 1 Neurophysiology, Behavior and Animal Welfare Assessment, DPAA, Universidad Autónoma Metropolitana (UAM), Mexico City, Mexico; 2 Department of Agricultural, Forestry, Food and Environmental Sciences, University of Basilicata, Potenza, Italy; 3 Department of Animal Science, Federal University of Rio Grande do Sul, Porto Alegre, Brazil; 4 Department of Animal Science, Federal University of Santa Maria, Santa Maria, Brazil; 5 Facultad de Estudios Superiores Cuautitlán, FESC, Universidad Nacional Autónoma de México (UNAM), Cuautitlán, Estado de México, Mexico; 6 Division of Neonatology, Hospital Infantil de México Federico Gómez, Mexico City, Mexico; 7 Departamento de Ciencias de la Alimentación, División de Ciencias Biológicas y de la Salud, Universidad Autónoma Metropolitana Unidad Lerma, Lerma de Villada, Estado de México, Mexico; 8 Programa de Ganadería, Colegio de Postgraduados-Campus Montecillo, Texcoco, Estado de México, Mexico; 9 Division of Biotechnology, Instituto Nacional de Rehabilitación Luis Guillermo Ibarra Ibarra (INR-LGII), Mexico City, Mexico; 10 Laboratory of Animal Health, LARSANA, Federal University of Western Pará, UFOPA, Pará, PA, Brazil

**Keywords:** calving, colostrogenesis, imprinting, lactation, sensitive period

## Abstract

The peripartum period is a critical biological stage characterized by coordinated endocrine, physiological, and behavioral adaptations that determine successful parturition, neonatal survival, and productive efficiency in water buffaloes. This review aims to discuss the changes that buffalo cows show at calving onset, as well as the implications of the mother–calf bond, lactation, and postpartum pathologies, highlighting their relevance to animal welfare and neonate viability. As in other ruminants, parturition is initiated by the activation of the fetal hypothalamic–pituitary–adrenal axis, triggering hormonal changes that manifest as non-invasive, field-applicable physiological and behavioral indicators useful for the early identification of the onset of parturition. After birth, the mother–calf bond is established during a brief (within the first 6 h), sensitive period through multisensory stimulation, primarily regulated by a complex neurological network modulated by glucocorticoids, hormones (e.g., oxytocin, prolactin), opioids, among others, which ensures selective maternal care and effective suckling. Lactation and immunoglobulin transfer through colostrum consumption are essential for neonatal survival, compensating for the minimal transplacental immunity. During the post-calving period, the dam’s health can be compromised by reproductive disorders such as placental retention or those related to the mammary gland (e.g., mastitis). A comprehensive understanding of these mechanisms supports strategies to optimize reproductive management, welfare, and neonatal viability and survival in buffalo production systems.

## 1 Introduction

Parturition is one of the most critical phases in the reproductive cycle of animals, including water buffaloes (*Bubalus bubalis*), involving endocrine, physiological, and behavioral adaptations that are essential for successful delivery and, consequently, neonatal survival (1–4). In buffalo, parturition is especially important due to its duration, the species’ unique anatomical characteristics, and the economic importance of production systems in both tropical and subtropical regions (5, 6). Any failure during this period compromises maternal well-being, increases the risk of dystocia, and negatively affects the calf’s viability and productive performance (7, 8).

In water buffaloes, prepartum behavioral changes such as increased walking, tail raising, ground pawing, abdominal-directed head movements, and reduced rumination have been quantified in Mediterranean and Murrah buffaloes. These behavioral changes have been identified as early indicators of calving onset (2, 3). Additional signs reported in the hours preceding parturition include restlessness, lateral tail movements, back arching, flexing of the hocks, and watery diarrhea (9, 10). In this context, comprehensive clinical, observational, and automated monitoring management has shown promising results, reinforcing its value as a practical tool. Immediately after calving, the mother–calf bond is usually consolidated during the first hours postpartum (11, 12), but its efficiency may vary with parity, management, environmental stressors, and early separation. This process relies on a complex integration of olfactory, tactile, auditory, and visual stimuli, which are processed by the limbic system and sensory cortices under the modulation of oxytocin (13–15). This ethological connection not only ensures mutual recognition but is also a prerequisite for the feeding phase; without a strong bond, the physical interaction necessary for suckling is compromised.

After calving, the mammary gland (MG) transcends its purely nutritional function to become the determining axis for the survival and immunological development of the neonate (16, 17). This process starts with colostrogenesis and the selective transport of essential immunoglobulins (Ig) for a species with synepitheliochorial placentation (18, 19). The viability of the calf depends not only on the biochemical richness of colostrum (20–22) and its dynamic transition to mature milk, but also on the efficiency of the neuroendocrine mechanisms of milk ejection and the behavioral capacity of the calf to establish an effective sucking reflex (23). Therefore, the study of the interaction between maternal physiology and the biological response of the calf is essential to understand the mechanisms that ensure the transfer of passive immunity and homeostatic success in the first hours of life (24, 25). Additionally, in the post-calving period, buffalo cows are susceptible to reproductive disorders (e.g., placental retention and metritis) and other disorders, such as mastitis, that might not only compromise the dam’s health but also neonatal viability (26, 27). This review aims to discuss the changes that buffalo cows show at calving onset, as well as the implications of the mother–calf bond, lactation, and postpartum pathologies, highlighting their relevance to animal welfare and neonate viability.

## 2 Parturition onset: endocrine, physiological, and behavioral modifications before calving

Normal or eutocic birth is a crucial period for the buffalo dam and the newborn calf, which incorporates endocrine, behavioral, physiological, and morphological modifications at calving (1–3). In multiparous water buffaloes, the duration of parturition ranges from 233 to 729 min (average of 509.41 ± 45.75 min), where 80% of births occurred between 6 p.m. and 6 a.m. (28). Similar parturition duration was reported by Modi et al. (29) in 15 buffalo dams in the rural region of Mhow, with an average duration of 504.52 ± 45.97 min. Calving is influenced by several factors, such as the duration of parturition, the condition and dimensions of the calf, as well as the anatomical characteristics of the dam.

**Figure 1:**
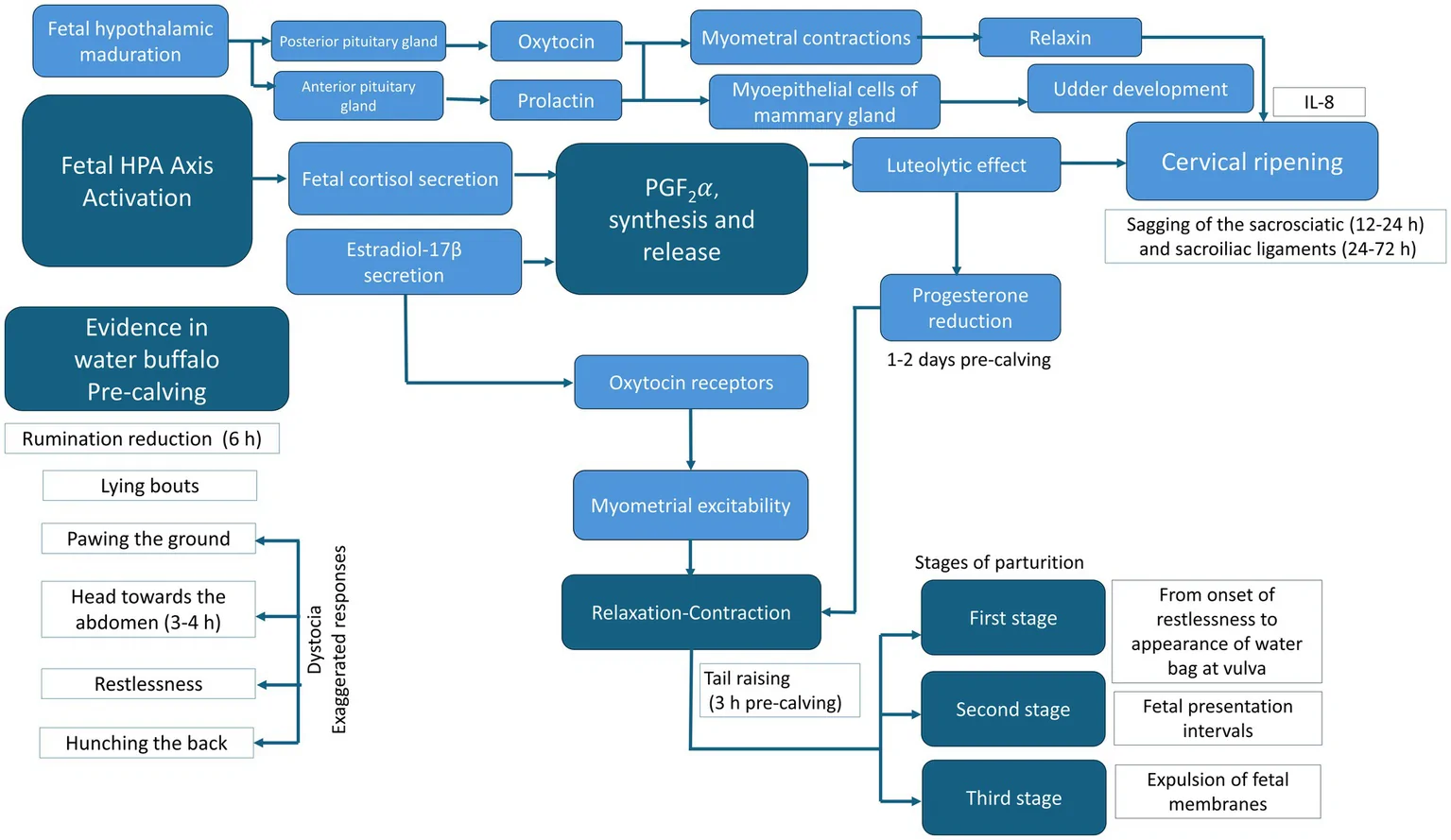
Physiological, behavioral, and endocrine mechanisms involved in parturition onset. Illustrates changes before calving and during parturition. HPA, Hypothalamic–Pituitary–Adrenal; PGF_2_α, Prostaglandin F_2_α.

Regarding the endocrine changes related to calving, it was reported in 3-year-old pregnant Murrah buffaloes under loose housing that progesterone (P_4_) levels decreased at the last stage of gestation, particularly 2 days before calving, reaching 1.34 ± 0.20 ng/mL (30). Concomitantly, circulating estradiol-17_β_ (E_2_) concentrations increased progressively during the 2 weeks preceding parturition (days 14 to 4 before calving) and reached a peak of 82.27 ± 9.49 pg./mL 1 day before calving (30). These findings are consistent with radioimmunoassay measurements in Murrah buffaloes (31), which showed an abrupt decline in P_4_ concentrations during the last 1–2 days before calving. This decline coincided with increased concentrations of PGF_2α_ and estradiol-17_β_ around parturition (Figure 1). In the same study, P_4_ concentrations were reported to be negatively correlated with PGF_2α_ (*r* = 0.70; *p* < 0.05), 13,14-dihydro-15-keto-PGF_2α_ (*r* = −0.83; *p* < 0.01), and estradiol-17_β_ (*r* = −0.89; *p* < 0.001) before calving (31). Similarly, Arora and Pandey (30) reported that circulating LH concentrations remained relatively stable throughout most of gestation, declined gradually during late pregnancy, reached their lowest values at parturition, and subsequently increased by post-calving day 16. Although the endocrine events preceding parturition have been extensively characterized in cattle, available evidence indicates that buffaloes exhibit a comparable prepartum decline in P_4_ accompanied by coordinated changes in estradiol-17_β_, PGF_2α_, and LH, although the endocrine mechanisms regulating the onset of parturition in *Bubalus bubalis* remain less comprehensively understood than in cattle.

Interleukin (IL) levels can also modulate parturition, as reported by Kumar et al. (32) in six Murrah buffalo dams evaluated during the peripartum period. It was found that plasma IL-8 levels were significantly lower during the pre-calving period (≤ 46.56 ± 14.02 pg./mL on pre-calving day 15 in spring, and ≤ 73.18 ± 18.56 pg./mL 1 day before calving during the hot-humid season). In general, plasma levels of IL-8 tended to be significantly higher during the hot-humid season (82.40 ± 6.60 pg./mL) in contrast to spring (58.21 ± 5.87 pg./mL). In addition, a correlation between IL-8 and the mean temperature and humidity index of the microclimate was reported (*r* = 0.25; *p* < 0.01). These results suggest that higher plasma levels of IL-8 during the hot-humid season may be related to heat stress. According to Singh et al. (33), this response can be mediated by the binding of the transcription factor HSF-1, activated by heat stress, to at least two heat shock response elements present in the IL-8 promoter. Additionally, the elevation of IL-8 in the pre- and post-calving stages may also be attributable to cervical ripening that facilitates calving, as has been characterized in dairy cows (34).

Eutocic births in water buffaloes can also be monitored according to the duration of each parturition stage and the events that occur during them. The first stage can have an average duration of 35.35 ± 1.08 min, while the second stage is 43.26 ± 1.21 min (28, 29). This stage is characterized by the onset of intense labor pain and the appearance of allantochorion, which can last 10.20 ± 1.04 min. Other events and their average duration are the appearance of allantochorionic and its rupture (2.33 ± 0.43 min), rupture of allantochorion and appearance of amniotic sac (7.90 ± 1.12 min), appearance of amniotic sac and appearance of fetal limbs (5.72 ± 0.80 min), appearance of fetal limbs and appearance of fetal muzzle (4.73 ± 0.33 min), appearance of fetal muzzle and appearance of fetal head (3.13 ± 0.45 min), appearance of fetal head and its expulsion (1.23 ± 0.47 min), expulsion of fetal head and appearance of fetal shoulders (2.83 ± 0.32 min), appearance of fetal shoulders and its expulsion (4.18 ± 0.47 min), and expulsion of fetal shoulders and expulsion of rest of the body. Finally, the third stage can have an average duration of 431 ± 46.42 min (28, 29).

In comparison, Deka et al. (35) studied the duration of the parturition stages of 30 swamp buffalo and the behavioral changes associated with these phases, reporting a reduction in parturition times. It was found that the onset of restlessness to the appearance of the water bag at the vulva began at 423.40 ± 130.57 min, the second stage at 29 ± 4.06 min, and the third stage at 233 ± 41.89 min.

Some signs of early calving include separation from the group, continuous standing postures, frequent tail movements, vocalizations, and sagging of the sacrosciatic and sacroiliac ligaments 12–24 h and 24–72 h pre-calving, respectively. In addition, significant changes in the development of the udder are evident and progressive in the last 7 days of parturition and extremely marked 3 days before calving, together with the distention and congestion of the teats 1.8 days pre-calving (36). This is related to uterine contractions and progressive cervical dilation between two and 3 days before calving (37).

A recent study by Rossi et al. (38) evaluated the degree of pelvic ligament relaxation, MG development, and softening of the cervical mucus plug as indicators to monitor the onset of calving in 15 Mediterranean buffalo heifers using an intravaginal device. The results activated an alarm 48 to 72 h before calving, enabling a preventative assessment of fetal presentation, position, and posture, along with the degree of cervical dilation. The mean duration of parturition was recorded (68 ± 9.8 min), which was significantly longer for male offspring compared to females (*p* = 0.002). In comparison, in dairy cattle (Holstein Friesian cows), bracelets (inclinometers and accelerometers) placed around the proximal part of the tail are currently used to record the frequency and duration of tail movements to monitor parturition onset (39). The results indicated an alarm report 24 h and 3 h before calving for all cows. Significant differences were shown in tail movements, where 8 h before calving, contraction of the tail increased (891 events). During calving, the tail remained relaxed for a shorter time (427 s/15 min), and the frequency and duration of tail raising were greater (3 events and 390 s/15 min, respectively). Eight hours before calving, the duration of lying down also increased (331 s/15 min), while eating behavior was absent (39).

It is important to consider that, during calving, physiological pain elicits changes, including restlessness, lack of appetite, and postural modifications, such as continuous shifts in lying down and standing up, tail raise, and rumination, as observed by Teja et al. (2) in Murrah buffaloes. Other behavioral changes, such as constantly looking at the flanks, head raising, scratching the ground, and tail raising, may be present during the contraction period before calving. Lanzoni et al. (3) evaluated lying behavior in 20 primiparous Italian Mediterranean buffaloes (*Bubalus bubalis*, subspecies *bubalis*) and reported an approximately 60% increase in lying-bout frequency, from 11.82 ± 2.48 to 19.82 ± 4.61 bouts during the 11-h control and pre-calving periods, respectively (*p* < 0.001). By comparison, lower lying-bout frequencies were reported 1 day before calving in multiparous buffaloes of other breeds, with mean values of 9.30 ± 0.24, 9.37 ± 0.20, 9.67 ± 0.26, and 9.77 ± 0.29 bouts/day for parity 2, 3, 4, and 5, respectively, indicating that parity may influence lying behavior during the prepartum period (40). However, lying-bout frequency did not differ significantly among parity groups (*p* > 0.05). In addition to changes in lying behavior, other prepartum behavioral activities became more pronounced as calving approached. Lanzoni et al. (3) reported that ground-pawing increased from 5.07 ± 4.98 during the control period to 20.29 ± 11.13 occurrences during the pre-calving period (*p* < 0.001). Likewise, head movements directed toward the abdomen increased from 15.06 ± 11.72 to 41.59 ± 19.29 occurrences (*p* < 0.001). Together, these behavioral changes may represent reliable indicators of imminent calving and are likely associated with the visceral discomfort caused by progressive uterine contractions and abdominal distension during parturition (41). Mohammad et al. (1) reported that the overall behavioral pattern did not differ significantly between buffalo heifers and multiparous buffalo cows. However, regardless of parity, animals experiencing dystocia exhibited a higher frequency of marked restlessness, pawing, looking back toward the abdomen, and back hunching than those undergoing eutocia (*p* < 0.01). Vocalization was more frequent in heifers than in multiparous cows (*p* < 0.05), whereas the frequency of lying down and standing up did not differ significantly between dystocia and eutocia. This has also been reported in Holstein dairy cows, where primiparous cows tend to exhibit more postural transitions than multiparous cows (42). Together, these findings suggest that behavioral changes are not isolated events but rather reflect the hormonal cascade that leads to the onset of calving, reinforcing their usefulness as non-invasive biological predictors.

**Figure 2:**
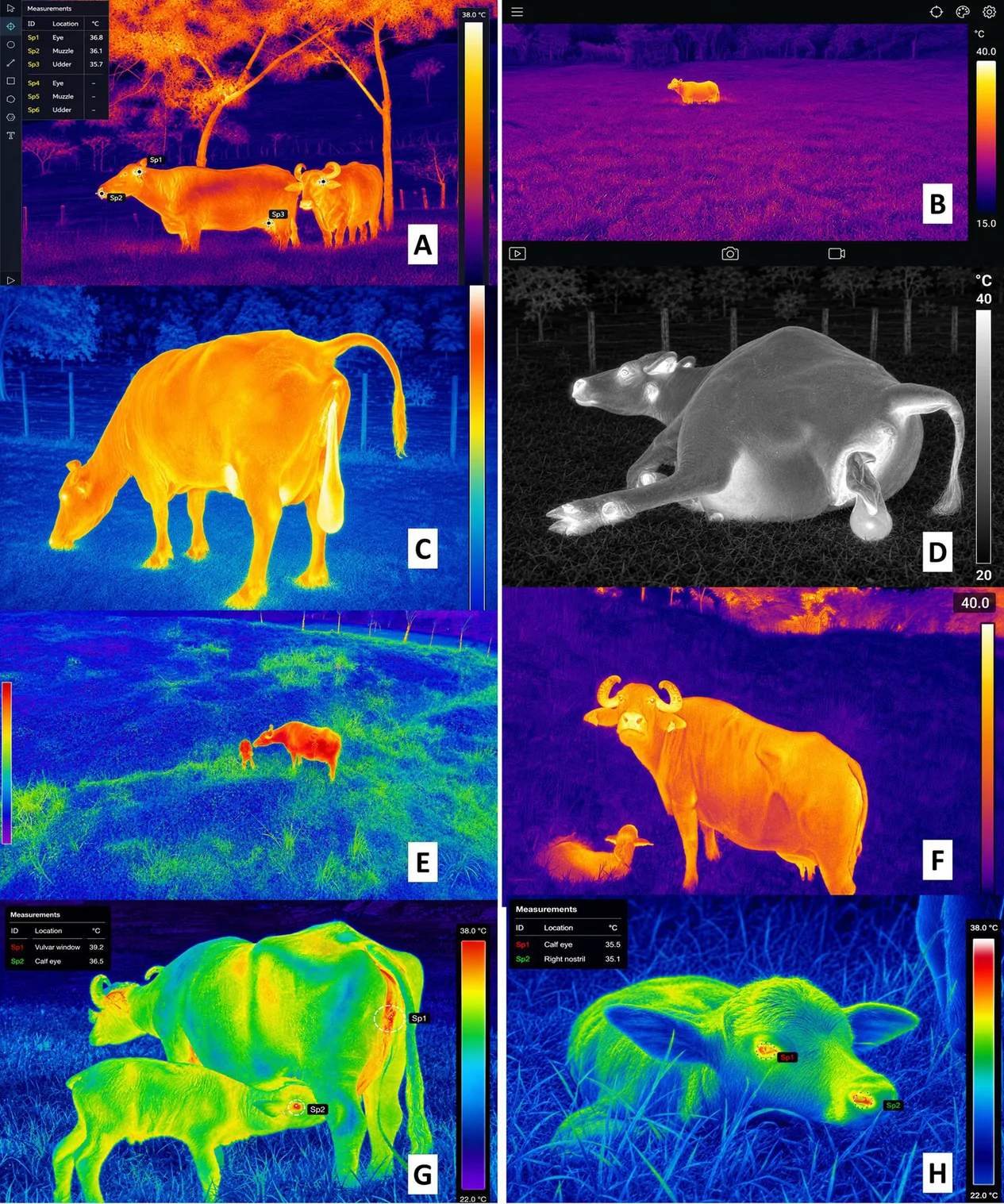
Artificial intelligence-enhanced representations generated from radiometric thermograms acquired with a FLIR E8® infrared camera during the periparturient period in water buffaloes (*Bubalus bubalis*) maintained under tropical pasture-based conditions. **(A–B)** Panoramic thermal monitoring of pregnant buffaloes approaching calving. **(C–D)** Thermal monitoring during the expulsive stage of nocturnal calving. **(E, F)** Maternal and neonatal thermal assessment immediately after birth. **(G)** Buffalo with retained fetal membranes at 36 h postpartum. **(H)** Calf with neonatal hypothermia showing ocular (Sp1) and right nostril (Sp2) thermal windows.

The prepartum behavioral signs described above, including tail movements, postural changes, restlessness, and altered locomotor activity, have been consistently associated with imminent calving in buffaloes (11, 12). In this sense, Teja et al. (2) evaluated the behavioral response of 26 multiparous Murrah buffaloes before calving (between parities three and seven). The results indicated that rumination time decreased from 165 ± 3.1 min/6 h at 12 h before calving to 127 ± 2.6 min/6 h during the final 6 h preceding parturition. A significant increase in activity was observed between six and 1 h before calving (9.5 ± 0.5 events). Changes in standing up/lying down increased by 3-fold in the last 6 h before calving (0.5 ± 0.5 events/6 h). Tail raising frequency increased from 1.69 ± 0.21 to 3.65 ± 0.31 between three and 1 h before calving, respectively. These behavioral modifications help to establish periods of rest, rumination, and tail raising, which can predict parturition in water buffalo dams. The increased restlessness and discomfort associated with calving were also reported by Miedema et al. (43) in multiparous dairy Holstein cows, using tail raising and duration of ground-licking. In primiparous Holstein cows, a decrease in core body temperature, between 0.5 and 1 °C at eight and 24 h before calving, was used to establish parturition onset using an intravaginal device (44, 45). Changes in the calving process require immediate attention, as dystocia is an obstetric emergency. In this regard, for Murrah Buffalo, recent records are based on retrospective studies, where uterine torsion is the most important cause of maternal dystocia (8) and case studies (7), where fetal causes are associated with improper position within the birth canal, with bilateral knee flexion and engagement of the forehead and pelvic brim. Taken together, all these endocrine, physiological, and behavioral changes constitute useful biological indicators for predicting the onset of labor, early detection of dystocia, and optimization of perinatal management. A comprehensive approach to these indicators not only improves mother–offspring welfare but also contributes to better reproductive decision-making and increased production efficiency in this species. Collectively, the endocrine, physiological, and behavioral modifications described above constitute a robust biological framework for predicting the onset of calving, facilitating the early recognition of dystocia, and optimizing periparturient management in water buffaloes. However, translating these biological indicators into practical decision-making under field conditions requires objective, continuous, and non-invasive monitoring approaches capable of assessing the physiological status of both the dam and the newborn. Accordingly, infrared thermography (IRT) has emerged as a valuable imaging modality for evaluating peripheral microcirculation through anatomically defined thermal windows, providing real-time information on thermoregulatory responses and physiological adaptations without the need for animal restraint or direct contact (46). Beyond its application to thermal stress assessment, IRT has enabled the characterization of ocular, nasal, vulvar., and mammary thermal windows in water buffaloes, where variations in surface temperature reflect vascular changes associated with thermoregulation, reproductive physiology, and health status (47). Moreover, integrating thermal imaging with current knowledge of buffalo thermobiology has expanded our understanding of the neurophysiological and behavioral mechanisms underlying adaptation to environmental challenges and the periparturient period (48). The clinical interpretation of infrared thermographic findings depends on the appropriate selection and validation of anatomical thermal windows, as well as on the physiological mechanisms regulating heat dissipation during health and disease. Consequently, standardized thermal assessment enhances the diagnostic value of IRT for monitoring thermoregulatory status and facilitating the early detection of inflammatory or febrile conditions in water buffaloes and other domestic species (49–51). As illustrated in Figures 2A,B, panoramic thermal monitoring of the herd may facilitate the identification of pregnant buffaloes approaching calving through increases of approximately 0.2–0.5 °C in the ocular, vulvar., and udder-base thermal windows, changes associated with the onset of the first stage of labor. During the expulsive stage, continuous thermographic surveillance may also be conducted under nocturnal conditions. Figures 2C,D shows the maintenance of physiological superficial thermal patterns during nocturnal calving, with temperature increases not exceeding 0.5 °C, while also illustrating the potential of IRT for detecting thermal alterations compatible with febrile processes during the periparturient period. Immediately after birth, simultaneous thermographic assessment of the dam and calf may provide information on maternal thermal homeostasis and neonatal adaptation. In Figures 2E,F, the observed thermal patterns were consistent with maternal normothermia and adequate neonatal thermal adaptation, with no thermographic evidence of alterations compatible with maternal fever or neonatal hypothermia. In addition, IRT may contribute to the early recognition of postpartum disorders. Figure 2G depicts a water buffalo diagnosed with retained fetal membranes at 36 h postpartum, in which a localized temperature increase was detected at the vulvar thermal window. This finding illustrates the potential of IRT as a non-invasive screening tool for postpartum alterations and may support timely veterinary intervention aimed at reducing the risk of vulvar., cervical, and uterine infections and subsequent puerperal complications, including metritis, mastitis, and agalactia.

Importantly, the application of IRT extends beyond maternal monitoring, as facial thermal windows provide a practical means for the early identification of neonatal hypothermia, enabling timely supportive interventions aimed at preserving neonatal viability and ensuring adequate colostrum intake (52). This approach is further supported by recent evidence demonstrating that neonatal surface temperatures are influenced by birth weight and time of day, reinforcing the value of thermal imaging for assessing thermoregulatory adaptation during the first days of life (53). Figure 2H shows a calf with neonatal hypothermia, characterized by superficial temperatures of 35.5 °C at the ocular thermal window (Sp1) and 35.1 °C at the right nostril (Sp2), together with poor vigor to stand and suckle. These findings support the potential use of IRT for the early identification of neonates with impaired postnatal adaptation and for the prompt implementation of supportive measures, including warming and ensuring adequate colostrum intake (Figure 2).

## 3 Cow–calf bonding: the sensory process post-calving

### 3.1 Neurophysiological and endocrine aspects

Cow–calf bonding for ruminants is a complex and essential biological process to ensure the protection, nutrition, and social development of the newborn calf (13, 54). However, most of the studies focused on cow–calf bonding neurophysiology have been performed in other species, such as cows, sheep, and goats (13, 55). Therefore, evidence in water buffaloes is still limited (56–58). This bond is established through reciprocal interaction during a limited period called the “sensitive period,” which begins immediately after calving and is estimated to last up to 6 h in ruminants such as Jersey and Friesian cows (11) and Maltese goats (12). During this stage, the dam’s neural plasticity may increase, allowing sensory cues from the calf to be consolidated and facilitating the establishment of exclusive maternal care (13, 59). Dam–newborn bonding involves neurobiological aspects that promote the transfer of passive immunity through colostrum intake. Moreover, establishing this bond tends to reduce neonatal mortality rates during the first few days after birth (60).

**Figure 3:**
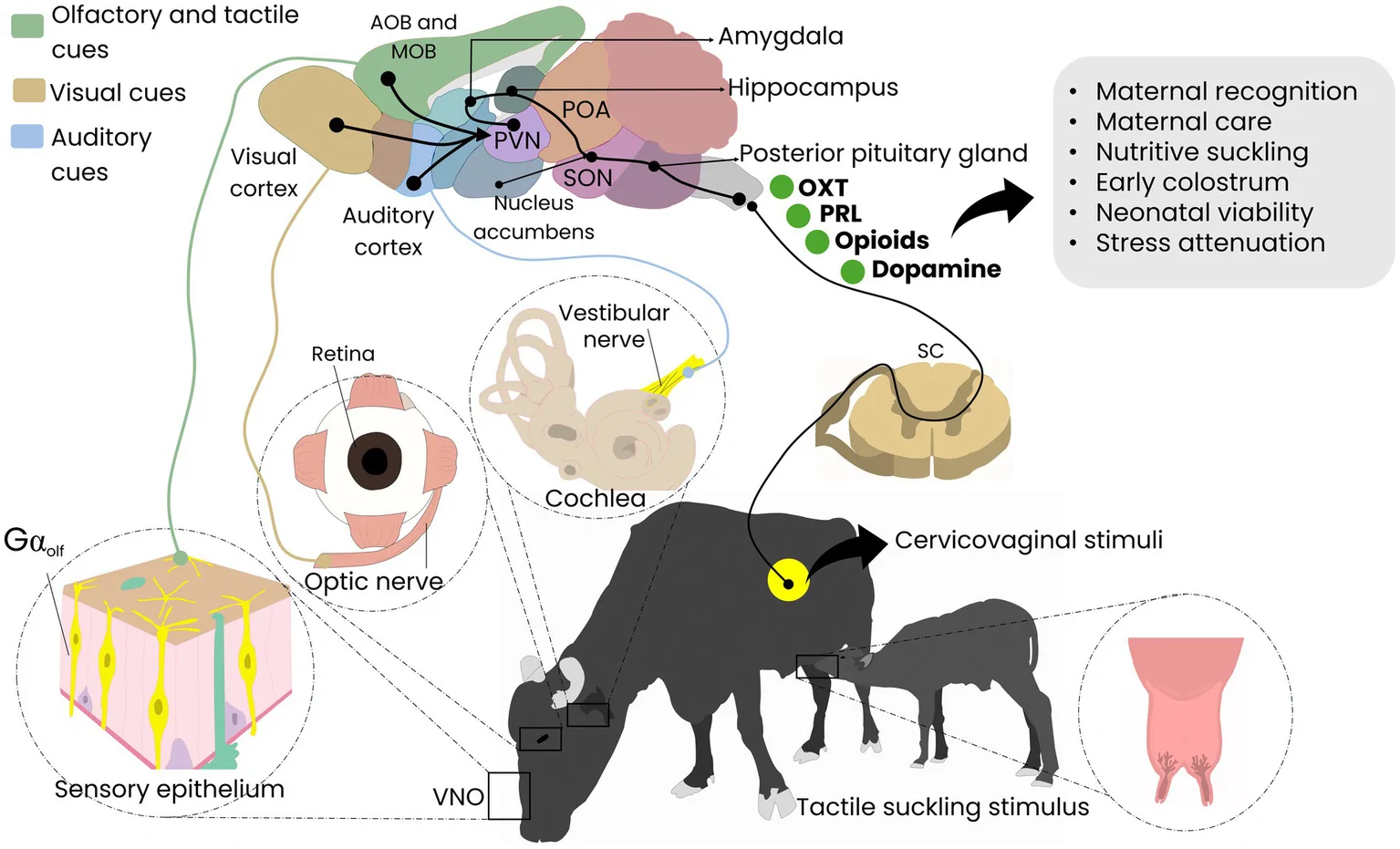
Multisensory neurobiology of dam–calf bonding in water buffaloes. During the sensitive period, the buffalo dam and the newborn interact through olfactory, visual, tactile, and auditory cues. Olfactory information detected by the vomeronasal organ (VNO), together with visual and auditory inputs, is integrated in brain regions involved in maternal behavior, including the medial preoptic area (POA), paraventricular nucleus (PVN), supraoptic nucleus (SON), amygdala, and hippocampus. Tactile stimulation through suckling and cervicovaginal stimulation further modulate this neuroendocrine network. POA, medial preoptic area; AOB, accessory olfactory bulb; MOB, main olfactory bulb; OXT, oxytocin; PRL, prolactin; PVN, paraventricular nucleus; SON, supraoptic nucleus; VNO, vomeronasal organ; SC, spinal cord; Gαolf, olfactory G-protein.

In ruminants, this process involves the interaction between sensory signals, behavioral responses, and neuroendocrine mechanisms that are activated during the sensitive period (15, 58). Figure 3 shows the sensory pathways and neurochemical mediators of maternal bonding (61–64). Specifically, according to the evidence in ewes and lambs, the establishment of dam–newborn bonding is mediated by a neuroendocrine system in which oxytocin, prolactin, glucocorticoids, dopamine, opioids, and other neurochemical mediators are essential to promote maternal behavior (64–66). During the expulsion phase of parturition, the newborn calf stimulates the cervicovaginal region, triggering the Ferguson reflex. Although the neuroendocrine mechanisms underlying maternal behavior have been well described in dairy cattle (67) and goats (68), comparable studies in water buffaloes remain limited. This mechanism involves the activation of nerve fibers that terminate in the medial preoptic area (MPOA) and the hypothalamus (69). This neuroendocrine pathway not only contributes to uterine contractions during parturition but is also involved in the activation of maternal behavior. Based on evidence from sheep and cattle, cervicovaginal stimulation activates oxytocinergic neurons located in the paraventricular (PVN) and supraoptic nuclei of the hypothalamus. These neurons release oxytocin centrally into brain regions such as the MPOA, a key center for the initiation and maintenance of maternal care and offspring recognition (64–66, 69). Using other ruminants as reference models, a significant increase in salivary oxytocin has been reported in Holstein cows on the day of calving, reaching up to 10.4 ng/mL (70). In pregnant Clun-forest ewes, cerebrospinal fluid oxytocin levels during lambing significantly increase to 76.5 pg./mL (71). As summarized in Figure 3, current knowledge of the neurobiology of maternal bonding in water buffaloes is largely inferred from studies in cattle, sheep, and rodents. Based on evidence from these species, Gα_olf_ located in the sensory epithelium of the VNO is thought to participate in the detection of chemical cues derived from the amniotic membranes and maternal odors, transmitting this olfactory information to the AOB and MOB. Olfactory, visual, auditory, tactile, and cervicovaginal stimuli subsequently converge on hypothalamic and limbic structures involved in maternal behavior, promoting the release of OXT, PRL, dopamine, and other neurochemical mediators that contribute to offspring recognition, maternal care, and the establishment of the dam–calf bond.

Despite the lack of studies directly evaluating the endocrine mechanisms underlying the activation of olfactory, tactile, visual, and auditory sensory systems during calving in water buffaloes, available evidence from other ruminants suggests that these pathways are modulated by activation of the oxytocinergic system together with coordinated neuroendocrine changes involving prolactin, P4, E2, glutamate, dopamine, and other mediators. In turn, this neuroendocrine milieu may facilitate long-term synaptic plasticity within brain regions involved in maternal behavior, including the olfactory bulb, amygdala, hippocampus, cerebral cortex, and bed nucleus of the stria terminalis, where sensory cues such as the odor of the fetal membranes are integrated to promote offspring recognition and affiliative maternal behaviors (72).

Building on the behavioral indicators outlined above, remote aerial observation may provide a complementary approach for monitoring water buffaloes managed under pasture-based conditions. By enabling wide-area assessment while potentially minimizing observer interference, drone-derived imagery can support the characterization of herd distribution, locomotor activity, social spacing, and the relative isolation of individual females. These variables may be particularly informative during the prepartum period, as withdrawal from the herd and alterations in activity patterns have been associated with imminent calving and may help identify animals requiring closer surveillance. During the immediate postpartum period, aerial observation may also facilitate the documentation of early maternal and neonatal responses, including the calf’s attempts to stand, dam–calf proximity, maternal attendance, and vigilance. Although these observations cannot replace direct clinical examination or detailed ethological assessment, they may provide complementary information on neonatal vigor and on the early interactions that precede udder seeking, colostrum intake, and the establishment of the dam–calf bond (73).

**Figure 4:**
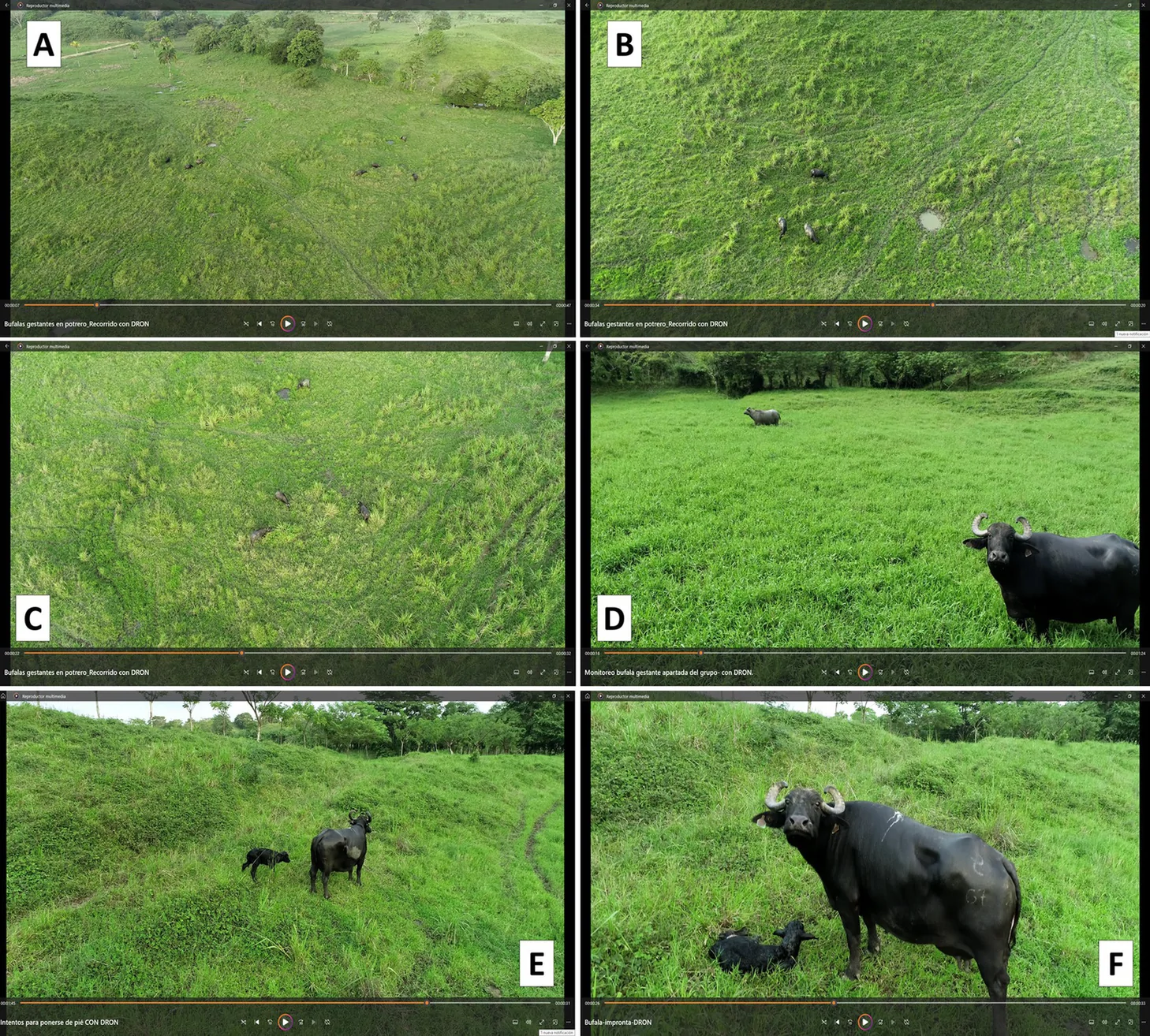
Aerial monitoring of periparturient behavior and early dam–calf interactions in water buffaloes maintained under pasture-based conditions. **(A–C)** Aerial assessment of herd spatial distribution, aggregation patterns, body orientation, interindividual distances, and the location of pregnant females within the paddock. **(D)** Pregnant female separated from the main herd, displaying behavioral signs associated with the first stage of labor, including restlessness, increased locomotor activity, repeated postural transitions, tail raising, and head movements toward the abdomen. When visible, pelvic ligament relaxation, vaginal discharge, and the appearance of fetal membranes or fetal limbs provide additional evidence of calving progression. **(E)** Newborn calf beside the dam, illustrating behavioral variables used to assess neonatal vigor. **(F)** Maternal attendance of a newborn calf during the early postpartum period.

As illustrated in Figures 4A–D, aerial monitoring enables the identification of behavioral and spatial patterns associated with the progression of parturition, including increased locomotor activity, repeated postural transitions, progressive separation from the herd, and changes in interindividual distance that may facilitate the recognition of females requiring closer observation. In addition, the visualization of pelvic ligament relaxation, vaginal discharge, or the appearance of fetal membranes and fetal limbs may provide complementary evidence of labor progression when image resolution and animal position permit.

Similarly, Figures 4E,F highlights the value of drone-based monitoring for assessing early neonatal adaptation and maternal behavior immediately after birth. Variables such as latency to stand, postural stability, locomotor activity, udder-seeking behavior, maternal attendance, vigilance, and dam–calf proximity may provide practical indicators of neonatal vigor and the establishment of the maternal bond under pasture-based conditions. Together, these observations support the use of aerial monitoring as a complementary tool for documenting behavioral events during the periparturient period while minimizing disturbance to the animals.

Accordingly, drone-based monitoring could contribute to a more integrated assessment of the transition from prepartum behavioral changes to early postpartum care under extensive management conditions; however, its predictive performance, measurement reliability, and operational feasibility in water buffaloes remain to be systematically validated (Figure 4).

### 3.2 Sensory mechanisms

#### 3.2.1 Olfactory and tactile stimulation

Olfactory contact and grooming are the most critical stimuli for ruminants. Intense sniffing and licking of the newborn calf begins from the cranial to the caudal region, culminating in the perineal area of the offspring (55). Grooming is considered the main sensorial mechanism by which the dam identifies the chemical signature of her offspring (72). Therefore, buffalo prioritize licking and standing over maintenance activities, such as feeding or lying down, during the sensitive period, as disruption of these early maternal responses may impair mother–young bonding and compromise neonatal care (74). This behavioral pattern has been described in primiparous Italian Mediterranean buffaloes continuously monitored by video during the first 48 h postcalving. During the first postcalving hour, dams spent 17.4 ± 9.5 min grooming their calves and 8.0 ± 6.7 min feeding. Grooming time declined significantly over the first 6 h postcalving (*p* < 0.001), whereas feeding time remained unchanged (*p* = 0.30). During the first postcalving day, dams devoted a total of 88.0 ± 25.0 min to calf grooming. Collectively, these findings indicate that maternal grooming predominates over maintenance behaviors, such as feeding, during the immediate postcalving period (3).

In gregarious species, such as water buffaloes, sniffing is the sensory precursor of licking, and the neuronal pathway is regulated by the vomeronasal organ (VNO). According to what has been reported in murine models in Sprague–Dawley rats (75) and in Dorsett ewes (61), the VNO of the dams retains a sensory epithelium replete with odor receptors that respond selectively to the odor of amniotic fluids. There is a bidirectional neuronal connection between the VNO and brain structures such as the accessory olfactory bulb and the main olfactory bulb, allowing the reception of information in brain centers such as the amygdala and the hypothalamus, which promotes cow–calf bonding (76–78). Furthermore, grooming complements placentophagy and is critical for newborn survival by drying the calf’s coat, encouraging the newborn calf’s respiration, and promoting the calf’s first defecation and urination (57, 58, 79). This has also been observed in cattle (*Bos taurus*), and it has been mentioned that this behavior can contribute to preventing postnatal hypothermia and stimulating peripheral circulation (10, 49, 80).

Research has shown that grooming and licking are highly influenced by parity (15). For example, multiparous parturient Murrah, Jaffarabadi, and Mediterranean buffaloes possess maternal memory that allows them to react more quickly to stimulate their offspring. They exhibit a significantly higher frequency (0.29 ± 0.06 episode/day) and duration (157.6 ± 27.8 s/day) of nursing behavior in the second and third parity than during the first calving (0.07 ± 0.06 episode/day and 25.9 ± 29.1 s/day, respectively) (81). In addition, multiparous Surti buffalo cows show a shorter latency from birth to suckling (often within the first 130.88 ± 7.81 min post-calving). In contrast, primiparous dams lack the previously established neural pathways. Primiparous dams could exhibit neophobia toward their own calf and can have a longer latency from birth to suckling (142.30 ± 10.66) (54, 57, 59). In some cases, the primiparous dam (Friesian cows) may even display escape behaviors such as kicking when the calves attempt to approach the inguinal area (56, 58, 82).

#### 3.2.2 Auditory stimulation

Buffaloes and their calves emit distinctive vocalizations. Vocal communication acts as an essential reinforcement of the olfactory bond during the mother–offspring period. A recent comparative analysis suggests that buffalo and cattle dams emit distinctive vocal patterns specifically designed to re-establish contact with their offspring after separation. Lenner et al. (83) identified significant differences between the vocal signatures of water buffalo cows and grey cattle cows subjected to a weaning stress situation (without physical contact only). The results showed that water buffalo dams emit lower-frequency calls (median 59.18 Hz) and grey beef cattle (*Bos taurus*) produce significantly higher-pitched vocalizations (median 95.6 Hz). Furthermore, they found that the buffalo’s vocalization had more dynamic pitch modulation—approximately three times more than that of the cows–, which could suggest a complex acoustic signaling system in buffalo during the mother–offspring period. These vocalizations, with their particular frequency and rhythmic structure, appear to act as an essential communication mechanism during mother–offspring isolation.

Additionally, reference data in *Bos taurus* cattle reaffirm the dynamics of acoustic recognition, where the dam of crossbred beef cattle uses low-frequency calls (81.17 ± 0.98 Hz) to call the calf to the udder during the first 3–4 weeks post-calving, while the calves use high-frequency calls (152.8 ± 3.10 Hz) when stressed (not in visual contact). This acoustic recognition is fully consolidated by 24–48 h postpartum (84). Thus, maternal and filial attachment mediated by auditory stimuli shows specialization according to the type of vocalization. As additional comparative evidence from small-ruminant models, it has been observed that in two-day-old lambs, low-pitched bleats are sufficient for individual identification of the mother. However, in the case of high-pitched bleats, the bond depends on vocal exchange. Under this mechanism, the lamb identifies the specific vocal response that the mother emits exclusively in response to her offspring (72). In fact, acoustic recognition of lamb by ewes at 2 weeks post-lambing is significantly higher (response score 8) for their own lamb, in contrast to 0–1 for alien lamb. Therefore, the establishment of the bond requires the maintenance of bidirectional auditory channels, allowing the vocalizations of the mother and offspring to reinforce each other and strengthen imprinting.

#### 3.2.3 Visual stimulation

For ruminants, the visual system is not the primary mediator of early recognition (54). However, the dam monitors the position and condition of her offspring, which is crucial for responding quickly to threatening situations. According to models in small ruminants, in Merino sheep, maternal visual recognition matures late and is established between the second and third week of life in newborn (85). It has even been shown that visual recognition of the offspring activates the mother’s temporal and frontal cortex with an intensity equivalent to that recorded during auditory stimulation by her own lamb bleats (72). This capacity for visual discrimination is fundamental for the stability of the bond once the reliance on olfactory (short-range) senses decreases in gregarious species such as water buffalo.

The dam-calf bond established immediately after birth is a complex multimodal and neuroendocrine process in which reciprocal interaction during the sensitive period guarantees mutual recognition. It is important to promote this interaction so the dam can differentiate her biological offspring and prevent rejection. In Holstein cows, it has been observed that the precocity of the bond requires physical contact of at least 5 min post-calving to be decisive and establish the consolidation of cow–calf bonding (11, 86). Conversely, if the individuals suffer a separation of 5 h, this interference, reported in Holstein cows, acts as a severe ethological disruptor, causing up to 50% of the females to vigorously reject their own calves and other offspring due to the failure of immediate sensory recognition (11, 87).

## 4 The post-calving period

The postnatal period is critical for both the buffalo dam and newborn calf, as it involves the immediate provision of colostrum and the dam’s endocrine stimulation for lactation (88, 89). These stages can be summarized as colostrogenesis, mammogenesis, and lactation (90). Colostrogenesis—also known as lactogenesis I—refers to the migration of Ig from maternal circulation to the MG (91). Is a process that has been hypothesized to begin during late pregnancy in bovines, approximately three to 5 weeks before calving, and ceases abruptly before calving (91, 92). Colostrum is known as the “initial mammary gland secretion provided during the first 24–48 h after calving”; it is the first source of nourishment for newborn mammals (91, 93). Colostrum differs from milk, as it mainly contains maternal Ig (IgG being a critical Ig for passive immunity) that is transferred through colostrogenesis (94).

### 4.1 Colostrogenesis: endocrine modulation and mammary epithelial changes

Colostrogenesis has been researched in several ungulate species, mostly dairy cattle, sheep, and goats (95, 96). These studies have elucidated that colostrogenesis is an endocrine-mediated process associated with increased plasma levels of E_2_ and declining levels of P_4_ during the pre-parturition period (97). During this phase, the later stages of pregnancy in mammals are associated with increase in circulating E_2_ and decrease in P_4_ (97). This has been reported in primiparous and biparous Murrah buffaloes by Batra et al. (31), who determined the concentrations of E_2_ and P_4_ 7 days before calving. It was found that E_2_ increased over the last 7 days before calving (mean concentration of 99.07 ± 5.25 pg./mL), with peak concentrations of 141 pg./mL 1 day before calving. In contrast, in the study, P_4_ concentrations gradually declined (mean concentrations of 0.38 ± 0.07 ng/mL), with an abrupt fall at 1–2 days before calving.

Although research directly associating colostrogenesis and hormonal changes in water buffaloes is scarce, in non-pregnant dairy cows (Holstein-Friesian), Stark et al. (95) induced colostrogenesis through E_2_ and P_4_ injections followed by dexamethasone administration. After 7–8 days of injections, mammary secretory IgG_1_ increased significantly to 17 mg/mL and reached 15 mg/mL a couple of days before milking. These results suggest an endocrine influence for transcytosis, a process required to transport IgG_1_ across mammary epithelial cells to bovine colostrum (18). Furthermore, prolactin might also be involved in colostrogenesis, as peripheral plasma prolactin concentrations significantly increase in Murrah buffaloes 7 days before calving, with peak concentrations 1 day before calving (685 ng/mL) (31).

**Figure 5:**
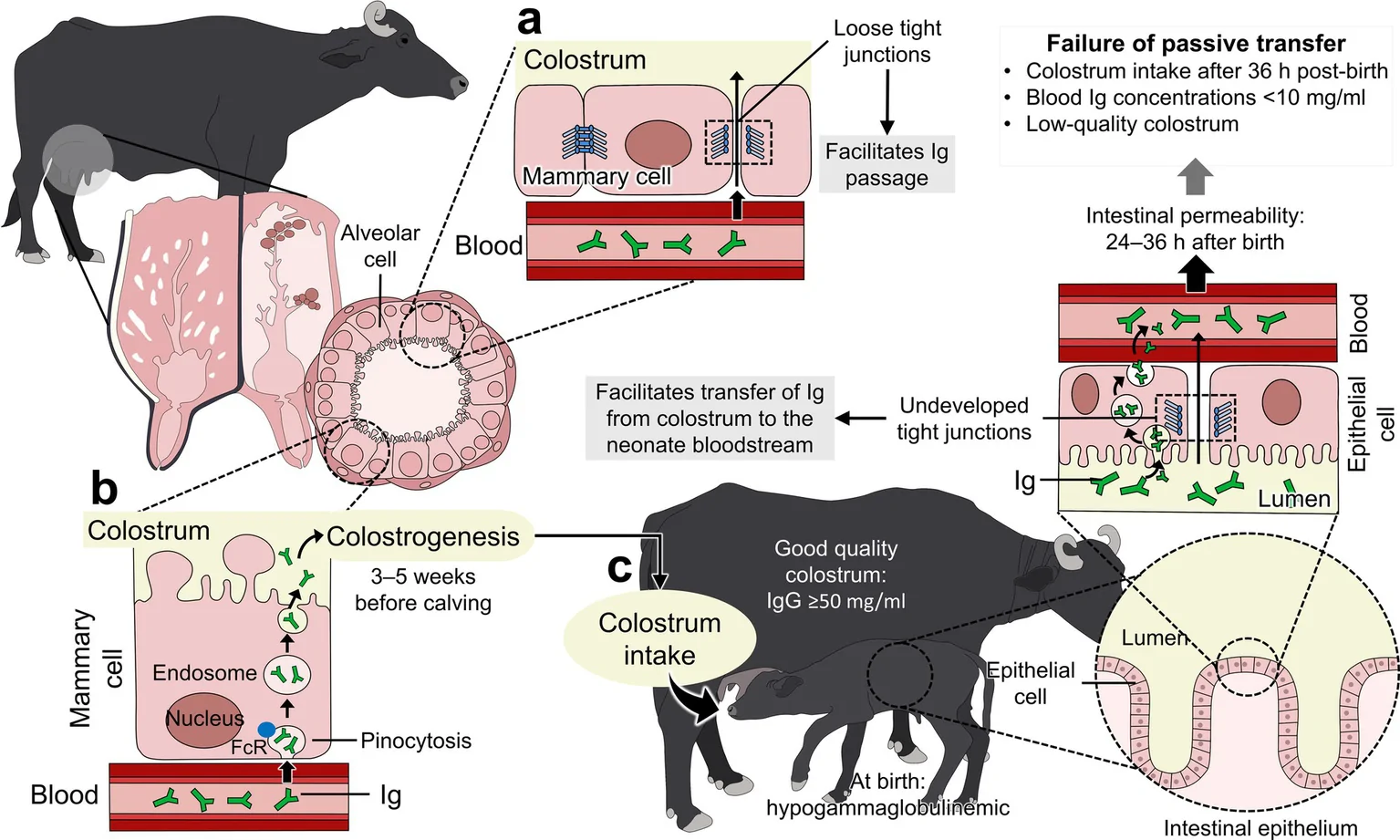
Colostrogenesis in water buffalo. Mechanisms and passive immune transfer in buffalo calves. **(a)** Transfer of Ig by the paracellular pathway. Loose tight junctions, located in alveolar cells, during gestation and the first days after calving, facilitate the passage of Ig from the blood to the colostrum. **(b)** Transcytosis mechanism of colostrogenesis. Circulating Ig can be transferred to the colostrum by pinocytosis and the participation of neonatal receptors, such as FcR. **(c)** Colostrum intake. The success of passive transfer requires the consumption of good-quality colostrum and the administration of colostrum within the first 24–36 h after birth. During this period, intestinal permeability is mediated by undeveloped tight junctions, facilitating the transfer of Ig from colostrum to the neonatal bloodstream.

Colostrogenesis is also a period that requires mammary epithelial re-structuration before calving (95, 98). During colostrogenesis, macromolecules (including antibodies or Ig) need to be transferred from maternal blood circulation or extracellular fluid to luminal secretions (Figure 5a) (18). In this sense, the mammary blood-milk barrier permeability in ruminants (as observed in cattle, ewes, and goats) is regulated by alveolar epithelial cells’ tight junctions (TJs), which are structures in the apical border of the cells (99, 100). During pregnancy, the mammary epithelium of species such as goats and cows is permeable, as these TJs are not fully developed before parturition. Therefore, the epithelium is still loose and allows the transfer of serum components (e.g., Ig) directly into the developing colostrum (101, 102). (Figure 5).

Mammary alveolar remodeling during pregnancy, lactation, and involution has been described in Murrah buffaloes. Chaurasia et al. (103) reported larger alveoli during late pregnancy, whereas involution was characterized by small, degenerative alveoli. In addition, both alveolar diameter and epithelial height differed significantly among physiological stages. Nevertheless, this study did not evaluate mammary epithelial TJs or Ig transfer. Consequently, evidence regarding mammary epithelial barrier function and Ig transfer in buffaloes remains limited and is largely extrapolated from studies in other ruminant species. Morgan and Wooding (104) evaluated the MG epithelium during pregnancy and lactation in Clun Forest and Soay pregnant ewes. In sheep, mammary epithelial TJs were significantly wider at 72 days of gestation than during late gestation, lambing, or established lactation. As pregnancy progressed, the junctions became narrower and exhibited an increased number of ridges (104). An increase in the number of TJs ridges has been associated with reduced paracellular ionic permeability, as proposed in experimental models of epithelial TJs (105). These findings suggest that changes in TJs permeability may contribute to the paracellular transfer of Ig before parturition. In dairy ruminants, the transition from colostrum secretion to copious milk production is associated with progressive closure of mammary epithelial tight junctions and restoration of the blood–milk barrier (106). This process has been morphologically demonstrated in ewes, in which TJ width decreased near lambing and during established lactation (104). Thus, although mammary alveolar histoarchitecture has been described in Murrah buffaloes, specific TJ remodeling and its relationship with Ig transfer during buffalo colostrogenesis remain insufficiently characterized.

Moreover, during colostrogenesis, mammary epithelial cells must be able to internalize IgG, transport it, and release it into the colostrum through receptor-mediated transcytosis (Figure 5b) (90). Although FcRn-mediated intestinal immunoglobulin absorption has been described in neonatal buffalo calves (107), studies directly characterizing FcRn expression and receptor-mediated IgG transport in the mammary gland of water buffaloes during colostrogenesis are still lacking. Therefore, evidence from dairy cattle is currently used as a comparative physiological model. In multiparous cows and heifers (*Bos taurus*), a study evaluated the mechanism of Ig transfer into the mammary secretion by comparing the concentration of IgG in blood serum and mammary secretion (92). It was found that while serum IgG concentration decreased significantly by 50% 2–3 weeks before calving, in mammary secretion, significantly higher IgG concentrations of up to 113.89 ± 11.41 mg/mL were observed in the same timeframe. These results show the transfer from blood into mammary secretion, potentially mediated by IgG-specific receptors located in mammary epithelial cells, such as the bovine neonatal Fc receptor studied in Holstein and Swiss Holstein-Friesian cows (108).

### 4.2 Maternal determinants of colostrum quality

Another aspect that influences passive Ig transfer success is colostrum quality, which is assessed according to the content of IgG, the most abundant Ig in bovine colostrum, accounting for over 75% (89, 109). In Murrah buffalo, Singh et al. (20) evaluated Ig levels (IgM, IgA, and IgG) in colostrum during the first hours after calving. The highest levels of Ig were observed during the first 4 h after calving (10.3 ± 4.9%). This percentage decreased to 8.2 ± 5.1, 5.1 ± 3.6, and 3.2 ± 2.0% at 16, 28, and 40 h post-calving, respectively (an event related to the transformation of colostrum into copious milk). The proportion of IgM, IgA, and IgG in buffalo colostrum reached up to 0.3, 0.7, and 98.4%, respectively, in the first hour post-calving. In the same breed, primiparous and multiparous Murrah buffaloes had a mean serum concentration of colostrum IgG of 71.4 ± 2.81 mg/mL (21). In primiparous Italian Mediterranean dams, colostrum IgG concentrations were 64.9 ± 29.3 mg/mL, and 77% of colostrum samples had IgG concentrations above 50 mg/mL (110). In other studies, at calving, the colostrum of Egyptian buffaloes registered IgG and IgM mean concentrations of 33.20 and 3.00 mg/mL, respectively (111).

Parity is considered one of the maternal factors influencing colostrum quality, as differences in Ig concentration have been reported among buffalo populations and physiological categories (21, 110, 111). Supporting this relationship, Souza et al. (112) evaluated neonatal Murrah buffalo calves born from dams of different parities and observed that maternal parity influenced serum globulin and IgG concentrations during the first month of life. Interestingly, calves born to primiparous buffaloes exhibited higher serum IgG concentrations after colostrum ingestion than calves born to multiparous dams, suggesting that parity may affect the efficiency of passive immunity transfer in buffaloes. In addition to parity, maternal management factors may influence colostrum quality and the success of passive immune transfer.

Evidence from dairy cattle indicates that colostrogenesis begins several weeks before calving and occurs predominantly during the dry period, when immunoglobulins are actively transferred from the maternal circulation to the mammary gland (113). Therefore, management practices during late gestation may affect both the quantity and quality of colostrum available to the newborn.

The duration of the dry period has been identified as one of these factors. In Holstein cows, omission of the dry period reduced colostrum production and decreased colostral IgG and IgM concentrations when compared with conventional dry periods of 30 or 60 days (114). Likewise, maternal nutrition during the dry period may affect colostrum composition. Supplementation with rumen-protected protein increased colostral IgG concentration and improved passive immunity transfer to calves, suggesting that adequate protein supply during late gestation supports colostrogenesis and neonatal immune protection (115).

Although the effects of dry-period length on buffalo colostrum characteristics remain poorly documented, prepartum micronutrient supplementation has been evaluated in Murrah buffaloes. Maternal supplementation with fat-soluble vitamins before calving increased colostral Ig secretion and modified serum Ig and micronutrient concentrations in neonatal calves, supporting a potential role in improving passive immune transfer and early postnatal development (116–118). Therefore, evidence from dairy cattle is currently used as the primary reference to understand the potential relationship between dry period management and colostrogenesis in this species.

Nevertheless, studies conducted in Murrah buffaloes indicate that dry period management influences several physiological and productive parameters that may indirectly affect maternal preparation for the subsequent lactation. Karthik et al. (119) reported that dry periods longer than 60 days were associated with greater postpartum body weight and increased milk yield in the subsequent lactation. Likewise, Reddy et al. (120) observed that shortened dry periods altered body condition dynamics and energy reserve mobilization during the transition period, while Reddy et al. (121) demonstrated effects of dry period length on productive and reproductive performance. Although these studies did not evaluate colostrum characteristics, they suggest that dry period management affects maternal physiological status during late gestation and early lactation, highlighting the need for future research investigating its direct effects on buffalo colostrogenesis and colostral Ig content.

Despite advances in characterizing colostral immunoglobulin secretion, neonatal Ig absorption, and factors influencing passive immunity in Murrah buffaloes, a validated species-specific threshold for classifying colostrum quality has yet to be established. Giammarco et al. (110) suggested that first-milking buffalo colostrum containing ≥50 mg IgG/mL, corresponding to approximately 18% Brix, may be considered high-quality colostrum. Similarly, Lotito et al. (122) highlighted the importance of providing colostrum with IgG concentrations above 50 mg/mL to ensure adequate passive immunity transfer in buffalo calves. For dairy cattle, colostral IgG concentrations of ≥ 50 mg/mL are considered good quality colostrum (123). Quality and the amount of colostral IgG decline rapidly after calving, as found by Dang et al. (124) in Murrah buffaloes when assessing IgG levels during the 5 days after calving. It was reported that immediately after calving, mean IgG levels were 54.0 mg/mL. However, they significantly decreased to approximately 50, 40, 30, and 18 mg/mL by day 2, 3, 4, and 5, respectively. These results highlight the importance of providing high-quality colostrum immediately after calving for two main reasons: because of the narrow period of increased neonatal intestinal permeability, and to prevent the decline in colostrum IgG content and quality.

### 4.3 Neonatal macromolecule absorption and gut closure

Immediately after birth, the provision of a timely supply of high-quality colostrum to newborn buffalo calves is critical for their survival (21, 110). In water buffaloes, studies have shown that Ig passage is limited across their placenta (20, 21). Therefore, neonate passive immunity transfer depends on colostrum intake to develop their own immunity as they are born hypogammaglobulinemic (Figure 5c) (21, 110). For instance, Souza et al. (21) determined serum IgG concentrations in male and female Murrah calves at birth and compared them to values after colostrum intake. At birth, the mean IgG concentrations were 4.23 ± 0.33 mg/mL. After colostrum intake, IgG levels significantly increased to 34.5 ± 1.48 mg/mL. In Murrah buffalo calves, Sikka and Sethi (125) reported a mean serum Ig concentration of 9.48 ± 0.41 mg/100 mL at birth. Similarly, Italian Mediterranean neonatal buffalo calves had mean IgG serum concentrations of 3.5 ± 0.4 mg/mL at birth. At 72 h after birth (after colostrum ingestion), IgG concentration increased significantly to 11.1 ± 2.0 mg/dL (110). Recent studies have proposed reference values for evaluating passive immunity transfer in buffalo calves. Giammarco et al. (110) reported similar serum IgG concentrations in healthy buffalo calves and proposed a serum Brix threshold of 8.4% for adequate passive transfer. Furthermore, Souza et al. (21) observed mean serum IgG concentrations of 34.5 ± 1.48 mg/mL at 24 h of age and considered passive immunity transfer successful in Murrah buffalo calves. Values below 10 mg/mL have also been associated with failure of passive transfer in buffalo calves, similarly to criteria adopted in cattle (126).

The success of passive Ig transfer depends on several factors, including the timing of colostrum intake (21), which is closely related to the physiological characteristics of newborn ruminants. Although fewer studies are available in water buffaloes than in dairy cattle, recent investigations have substantially improved our understanding of passive immunity transfer, colostrum quality, and Ig absorption in this species (21, 107, 110, 122). Similar to other ruminants, buffalo calves are born agammaglobulinemic or hypogammaglobulinemic and depend on the timely ingestion and absorption of high-quality colostrum to achieve adequate passive immunity transfer (21, 122). Moreover, Agrawal et al. (107) highlighted the importance of FcRn-mediated IgG transport in neonatal buffalo calves and reported that maximum Ig absorption occurs during the first hours after birth, reinforcing the importance of early colostrum feeding. This phenomenon is commonly referred to as the period of intestinal permeability to macromolecules or “open gut,” during which Ig can be absorbed through the intestinal epithelium before gut closure occurs (18, 127). In buffalo calves, efficient passive immunity transfer depends on the rapid uptake of colostral Ig during this limited postnatal window, a process mediated by intestinal epithelial transport mechanisms, including FcRn receptors (107). This transient permeability facilitates the transfer of Ig from colostrum into the neonatal circulation through the intestinal wall (128).

The buffalo calf’s absorption capacity of Ig can be determined by assessing its blood concentrations. It has been reported that high serum concentrations of IgG can be associated with colostrum intake within the first 4 h after birth (21). To investigate the *in vivo* percentage of absorption of colostral Ig from the intestine of a female Murrah buffalo calf, Singh et al. (20) collected precolostral blood plasma at birth, after colostrum intake, and at 8, 14, 15, 16, 31, and 34 h after birth. Before colostrum intake, the buffalo calf had significantly lower blood plasma Ig (0.94 g/100 g). After colostrum intake, blood Ig absorption reached 4.6 g/h at a percentage of 75.4%/h within 1 h after colostrum was fed. These results suggest that the newborn intestine can efficiently absorb Ig and macromolecules within the first 7 h after birth. However, this capacity decreases significantly after the first intake (to 0.83 g/h) (20). Moreover, the proportion of blood IgG increased after colostrum was fed, which suggests a preferential absorption of IgG.

Not providing high-quality or timely colostrum results in failure of passive transfer and may compromise the health of newborn buffalo calves. For instance, in Nili-Ravi buffalo calves, blood Ig concentrations below 0.41 g/dL, at 24–36 h after birth (after colostrum intake), were related to morbidity and mortality percentages of 66.7 and 36.7%, respectively (129). In contrast, buffalo calves with circulating Ig concentrations above 1.7 g/dL significantly reduced morbidity and mortality to 10.0 and 4.8%, respectively. Similarly, Okuyucu and Erdem (130) associated IgG concentrations of male and female Anatolian buffalo calves at 24–48 h post-birth with morbidity and mortality. It was found that buffalo calves that had circulating IgG concentrations below 10 mg/mL during the first postnatal day had a higher prevalence of diarrhea. This is relevant, as neonatal calf diarrhea in buffaloes causes the highest morbidity (16.6%) and mortality (5.2%) in Nili-Ravi buffalo calves in Pakistan (129). Moreover, low circulating concentrations of Ig (IgG, IgA, and IgM) can also increase calf mortality between birth and 180 days post-birth, as reported in Anatolian buffalo calves (130). Thus, ensuring colostrum intake within the first post-calving hours is recommended in water buffalo calves to promote passive immune transfer and reduce mortality risk.

### 4.4 Milk ejection and neonatal suckling behavior

Milk ejection is a neurophysiological event in mammals mediated by endocrine changes and tactile inputs from the MG, where suckling stimulus triggers a positive feedback loop (88). In primiparous and multiparous water buffaloes, approximately 90–95% of milk is stored in the alveolar fraction of the udder (131), and this amount is only available after milk ejection induced by oxytocin and myoepithelial contraction (17, 132). Although there are no reports in water buffalo, in multiparous Holstein cows, significantly higher plasma oxytocin concentrations were found during suckling events (above 250 pg./mL), in contrast to the period before or after suckling (approximately 180 pg./mL) (133). Similarly, in primiparous and multiparous Brown Swiss dairy cows, suckling increased plasma oxytocin concentrations up to 43.4 pmol/L (134). These results indicate the association between calves’ suckling behavior and the possible endocrine response that could also be present in water buffaloes.

Suckling behavior is considered a type of tactile cue that can directly stimulate peripheral receptors located in the mother’s nipples. In Italian Mediterranean calves, it has been reported that newborns attempt to suckle as soon as 48.8 min after birth, but have a mean latency of 212.0 ± 110.0 min between birth and their first successful suckling (58). In Surti buffalo calves, a shorter birth to suckling time was found, with 134.54 ± 6.28 min (57). Additionally, the authors also reported the duration of active teat seeking (71.74 ± 5.20 min) and the average time the calves delayed from locating to teat to suckling (13.00 ± 3.02 min). As discussed in the section regarding cow–calf bonding, promoting the interaction between the dam and the newborn immediately after calving is essential to initiate maternal behaviors, but also to promote suckling and, consequently, the milk ejection reflex.

Research regarding the milk ejection reflex has been mostly conducted in ewes and dairy cattle. However, these studies have found that oxytocin release from the posterior pituitary gland is in response to tactile teat stimulation by the neonate through suckling (135). For example, in non-lactating *Ovis aries* ewes, Tsingotjidou and Papadopoulos (132) determined the anatomic organization of primary afferent neurons in the nipples of the lamb. The authors reported that nipples are innervated by lumbar spinal ganglia (L2–L5). In progressive studies by the same authors in adult non-lactating, maternally experienced Chios and Karagouniki sheep, the spinal and supra-spinal pathway of the milk ejection reflex was established using tract-tracing techniques (136). In the study, the ascending neuronal pathway started with afferent inputs on the nipples projecting to the neurons of the L2–L5 of the spinal ganglion. These are transmitted to the laminae I–III of the L3 and L4 spinal segments, to the lateral cervical nucleus, the cuneate nucleus, and the PVN of the hypothalamus. From this center, a descending vascular link involves oxytocin release in the bloodstream and its action on the myoepithelial cells of the MG (137).

Although neurophysiological studies have yet to be performed in water buffalo species, the evidence published in other ruminants (e.g., dairy cattle and ewes) might serve as a basis to understand milk ejection in buffaloes, as schematized in Figure 5.

### 4.5 Post-calving disorders

#### 4.5.1 Placental retention and metritis

The period after calving, where uterine involution and hormonal balance are re-established, is crucial for the reproductive cycle of buffalo dams (138). However, different disorders might arise at this period. Recent studies have demonstrated that metabolic alterations associated with postpartum disorders may originate during the weeks preceding calving. In dairy buffaloes, prepartum non-esterified fatty acids (NEFA) concentrations above 0.29 mEq/L were associated with greater postpartum disturbances in energy metabolism, oxidative balance, inflammatory markers, and mineral status (139). Therefore, assessment of NEFA during late gestation may help identify animals requiring closer monitoring during the transition period. One of these disorders is placental retention (PR), a pathological reproductive condition where the placenta or fetal membranes are retained for more than 12 h after calving in water buffalo dams (140, 141). This condition is associated with mechanical difficulty in expelling fetal membranes that have already been detached, due to dystocia or insufficient uterine contractions (uterine inertia and atony) (142). Other authors highlight the influence of hormonal disorders involving estrogens and P_4_ (143).

In buffalo farming, PR is a costly complication that increases the susceptibility to endometrial sepsis, metritis, pyometra, cystitis, peritonitis, vaginal prolapses, and decreased milk production (144–146). Buffaloes diagnosed with PR are predisposed to uterine inflammation and infections due to the contamination of fetal membranes with feces and microorganisms, which has been associated with toxic puerperal metritis in 52.4% of buffaloes presenting PR (27, 141). Moreover, it delays uterine involution, increases the calving interval and number of services, and decreases buffalo fertility indices (26, 146).

The incidence of PR in water buffaloes ranges from 3 to 35.8% (26, 143). In Murrah buffaloes, the reported incidence was 2.89 and 9.67% (143), while in Egyptian buffaloes, the incidence has been reported at 19.04% (141). These percentages can be influenced by parity, where multiparous buffaloes above the fifth parity have recorded the highest incidence of PR (30%) (26, 143). Furthermore, when comparing the incidence of PR between water buffaloes and Iraqi cows, the incidence was significantly higher in buffaloes (up to 59.3%), while Iraqi cows recorded up to 40.7% (147). Other authors, such as Gupta et al. (143), reported that PR cases are also influenced by the season, where 50% of PR incidences in Murrah buffaloes were found in the rainy season, followed by 32% in winter, which might be related to higher rainfall, humidity, and thermal stress in hot-humid climates. In the same study, from 3,814 calvings, 5.24% of buffalo presented PR, and 60.57% of cases were associated with male calves.

Hormonal imbalances in water buffaloes have been associated with PR. For example, Elsayed et al. (26) evaluated the histopathological and hormonal profile of multiparous Egyptian buffaloes that expelled their placenta after 12 h post-calving. When comparing buffaloes with PR and healthy ones, PR animals had significantly higher serum P_4_ (approximately 2.0 *vs*. 1.2 ng/mL), cortisol (up to 60 *vs*. 20 ng/mL), and lower estrogen levels (approximately 100 vs. 350 pg./mL). Similar results have been reported by Atallah and Moustafa (148), who analyzed blood and placental tissue from 15 multiparous buffalo cows with PR. When compared to healthy animals, those with PR had significantly lower E_2_ concentrations (258.4 ± 32.7 *vs*. 380 ± 38.9 pg./mL) and higher cortisol values (1.4 ± 0.4 *vs*. 2.7 ± 1.2 ng/mL). In another study, the hormonal profile of Egyptian buffaloes diagnosed with PR revealed a significantly lower serum E_2_ concentration (36.73 ± 5.50 *vs*. 94.30 ± 3.21 pg./mL) and significant increases in P_4_ (9.13 ± 0.15 *vs*. 6.73 ± 0.38 ng/mL) (141). Increased P_4_ concentrations enhance uterine quiescence, while a reduction in E_2_ directly declines the production of PGF2α, leading to the suppression of myometrial contractions to expel fetal membranes after calving (149, 150). Thus, these findings suggest that peri-partum E_2_ deficiency contributes to PR in water buffaloes (26).

In Egyptian buffaloes, animals with PR had higher parities (6.9 ± 0.4), longer calving intervals (440.9 ± 20.9 days), and lower conception rates (57.14%) than healthy buffalo cows (5.6 ± 0.3, 402.4 ± 7.6 days, and 86.66%, respectively) (26). These conditions were associated with metabolic and oxidative stress, reflected in a significant reduction in superoxide dismutase (SOD) activity and increases in malondialdehyde (MDA) before and at calving. However, findings regarding total antioxidant capacity (TAC) in buffaloes with PR are not entirely consistent across studies. Atallah and Moustafa (126) reported significantly higher serum TAC concentrations in buffaloes diagnosed with PR (9338.0 ± 2372.04 mmol/L) compared with healthy buffalo cows (6226.9 ± 3568.3 mmol/L). This increase may reflect a compensatory antioxidant response to the heightened oxidative stress observed in affected animals. In Egyptian buffaloes with 24 h of PR, the activity of SOD and TAC of affected buffaloes significantly diminished by 59.5 and 75.4%, respectively, while serum MDA and nitric oxide (NO) significantly increased by ~3.8 and 1.3-fold, respectively (141). Similarly, in the same breed of buffaloes with PR, increased values of MDA (2.27 ± 0.44 *vs*. 0.98 ± 0.09 mmol/mL) and NO (22.29 ± 2.17 *vs*. 15.55 ± 1.58 μmol/L), and lower values of SOD (271.0 ± 17.39 *vs*. 338.16 ± 7.11 U/mL) were found (140). A deficient antioxidant system and increased production of reactive oxygen species are considered risk factors for PR since they can alter the concentration of steroid hormones and prostaglandins, necessary for the normal physiological process of calving (148). In dairy cattle, oxidative stress during parturition can affect uterine function by reducing phagocytic activity, resulting in failed placental detachment (151). Furthermore, buffaloes affected by placental retention also exhibit significant alterations in energy metabolism. Egyptian buffaloes that subsequently developed retained fetal membranes presented consistently higher concentrations of NEFA (0.7–0.8 *vs*. 0.5 mEq/L) and *β*-hydroxybutyrate (BHBA; 58.4–80.8 *vs*. 37.7–46.3 mg/dL) during the prepartum period and at calving when compared with buffaloes that expelled the placenta normally (26). These findings suggest that disturbances in energy metabolism and a more pronounced negative energy balance may contribute to the pathophysiology of placental retention and support the use of metabolic biomarkers as practical monitoring tools during the transition period. Moreover, it can activate inflammatory pathways that promote the release of inflammatory cytokines that impair uterine contractions to expel fetal membranes (152).

The metabolic and immunological changes in Egyptian buffaloes have also been investigated by Hassan et al. (141). Gynecological examination of animals affected by PR showed the presence of endometritis with bloody, purulent discharge. A significant increase of serum total cholesterol (114.23 mg/dL *vs*. 82.54) and triacylglycerol (141.43 *vs*. 93.79 mg/dL) was reported in affected buffalo cows, while high-density lipoprotein-cholesterol was reduced by 42.9% when compared to healthy buffaloes (49.86 *vs*. 87.32 mg/dL). The presence of hyperglycemia and hyperlipidemia in buffaloes with PR has been associated with insulin resistance or cortisol increases at calving (26, 153). Furthermore, in Egyptian buffaloes with PR, the expression of pro-inflammatory cytokines, such as interleukin (IL) 1β and IL-6, significantly decreased by 80.9 and 9.6%, respectively (141). Both cytokines have an essential role during the normal process of calving by activating prostaglandin pathways to stimulate myometrial contractions. Thus, the authors suggest that alterations in the immune response during calving might be the cause of PR.

From a practical standpoint, these findings suggest that monitoring metabolic and inflammatory biomarkers during the transition period may help identify buffaloes at increased risk of placental retention before the appearance of clinical signs. Early detection of metabolic imbalance could support preventive interventions aimed at improving postpartum reproductive performance.

The erythrogram, leukogram, and trace elements of Egyptian buffalo cows diagnosed with PR have also been evaluated (140). Of 813 buffaloes, 3.3% presented PR, and rectal palpation revealed that 100% of these animals had chronic endometritis with fibrosis. The erythrogram of buffaloes with PR had significantly lower red blood cells (4.21 ± 0.11 *vs*. 5.57 ± 0.11 × 10^6^/mL^6^) and hemoglobin (11.51 ± 0.28 *vs*. 14.90 ± 0.34 g/dL). These results revealed anemia with leucocytosis (8.24 ± 0.9 *vs*. 6.07 ± 0.36 10^3^/mL^3^), lymphopenia (53.42 ± 0.29%), and monocytosis (3.45 ± 0.26%), which is associated with an inflammatory response at calving in animals with PR (154, 155). Additionally, zinc, copper, iron, and selenium concentrations in PR buffaloes were significantly lower by 20.18, 9.29, 27.43 μg/dL, and 16.3 μg/L, respectively, elements that are required for the adequate function of the antioxidant system. Therefore, nutritional programs designed to maintain adequate trace mineral status during the last weeks of gestation may contribute to preserving antioxidant defenses and reducing the susceptibility of buffaloes to retained fetal membranes and associated uterine diseases.

According to the studies presented, PR is an important post-calving disorder that significantly affects buffalo health and is closely related to other pathological conditions, such as uterine infections. Entry of pathogenic bacteria leads to puerperal metritis, which is the inflammation of the uterus around 1 month of calving (156). The incidence of post-calving uterine infections in water buffaloes, including endometritis and metritis, ranges from 24 to 47.9% (138). When comparing the incidence of metritis between water buffaloes and Iraqi cattle, buffaloes recorded significantly higher percentages of metritis (63.1%) than cows (40%) (147). Among the risk factors associated with puerperal metritis, PR is one of the main causes. This was evaluated in Murrah buffaloes by Kumari et al. (157). In the study, it was found that the odds of metritis in buffaloes increased significantly with PR (odds ratio, OR = 125.42), abortion (OR = 11.87), shorter gestation length (OR = 6.57), and dystocia (OR = 6.12). Similarly, Azawi et al. (138) determined that Iraqi buffaloes with puerperal metritis had a clinical history of obstetrical problems, including PR (25%) and uterine/vaginal prolapse (7%), among others. In the same breed, 52.4% of buffalo cows diagnosed with puerperal metritis had a clinical history of PR (27).

During the post-calving period, the uterus of buffalo cows affected by PR is susceptible to colonization by both aerobic and anaerobic microorganisms, initiating an inflammatory response (138). Studies have reported that the predominant bacteria in lactating Iraqi buffaloes diagnosed with postpartum metritis were *Escherichia coli* (26.27%), *Klebsiella pneumoniae* (16.10%), *Lactobacillus acidophilus* (13.56%), and *Pseudomonas aeruginosa* (10.17%) (138). In the same study, the most prevalent kind of inflammation of the uterus was the chronic type (58%), characterized by atrophy of endometrial glands and fibrosis. Some authors have suggested that initial colonization of *E. coli* favors contamination with other microorganisms (27). A higher prevalence of *E. coli* was found by Verma et al. (156), who evaluated Murrah buffaloes that calved approximately 1 month before. The authors found that *E. coli* (66.7%) and *Staphylococcus aureus* (44.4%) were the main bacteria isolated from buffaloes with puerperal metritis. In Iraqi buffaloes with metritis, Azawi et al. (27) reported that the most prevalent bacteria in the uterine lumen were *E. coli* (18.5%), *Arcanobacterium pyogenes* (16.7%), *S. aureus* (13%), and *Fusobacterium necrophorum* (9.3%). Furthermore, the affected buffalo cows had a higher percentage of polymorphonuclear leukocytes (68.8 ± 6.3%) than healthy animals (31.7 ± 5.7%). Due to the presence of several bacteria, pharmacological treatment is necessary in buffaloes with puerperal metritis. Although there are studies where antibiotics such as rifampicin and oxytetracycline are an option to treat animals with a sensitivity of 73.8 and 67.9%, respectively (158), this might represent an economic constraint for buffalo farming. Thus, preventive measures to diminish the risk of infection (e.g., hygiene) are one of the main recommendations in buffalo husbandry.

The clinical signs associated with puerperal metritis are the response to several bacteria that have been isolated from the uterus. Buffaloes with puerperal metritis are characterized by mucopurulent vulvar discharge, pyrexia, anorexia, depression, and low milk yield (138, 156, 158). Animals might also present a staggering gait, paresis, and recurrent painful straining (27, 158). A study performed in Murrah buffaloes determined that animals with puerperal metritis presented watery fetid white or brownish discharge, and 88.89% of buffalo cows had an enlarged cervix (more than 7.5 cm) (156). Physiological variables reported pyrexia (rectal temperature of approximately 39.8 °C) and increases in pulse and respiration rate (110.4 ± 6.66 pulses/min and 46.2 ± 3.8 respirations/min, respectively). Pyrexia was also reported in Iraqi buffalo cows when compared to healthy buffaloes (40.9 ± 0.2 *vs*. 38.1 ± 0.1 °C) (27). Moreover, in these animals, 78.6% of buffaloes had a cervical diameter >7.5 cm with enlarged, asymmetrical horns, and uterine atonia.

**Figure 6:**
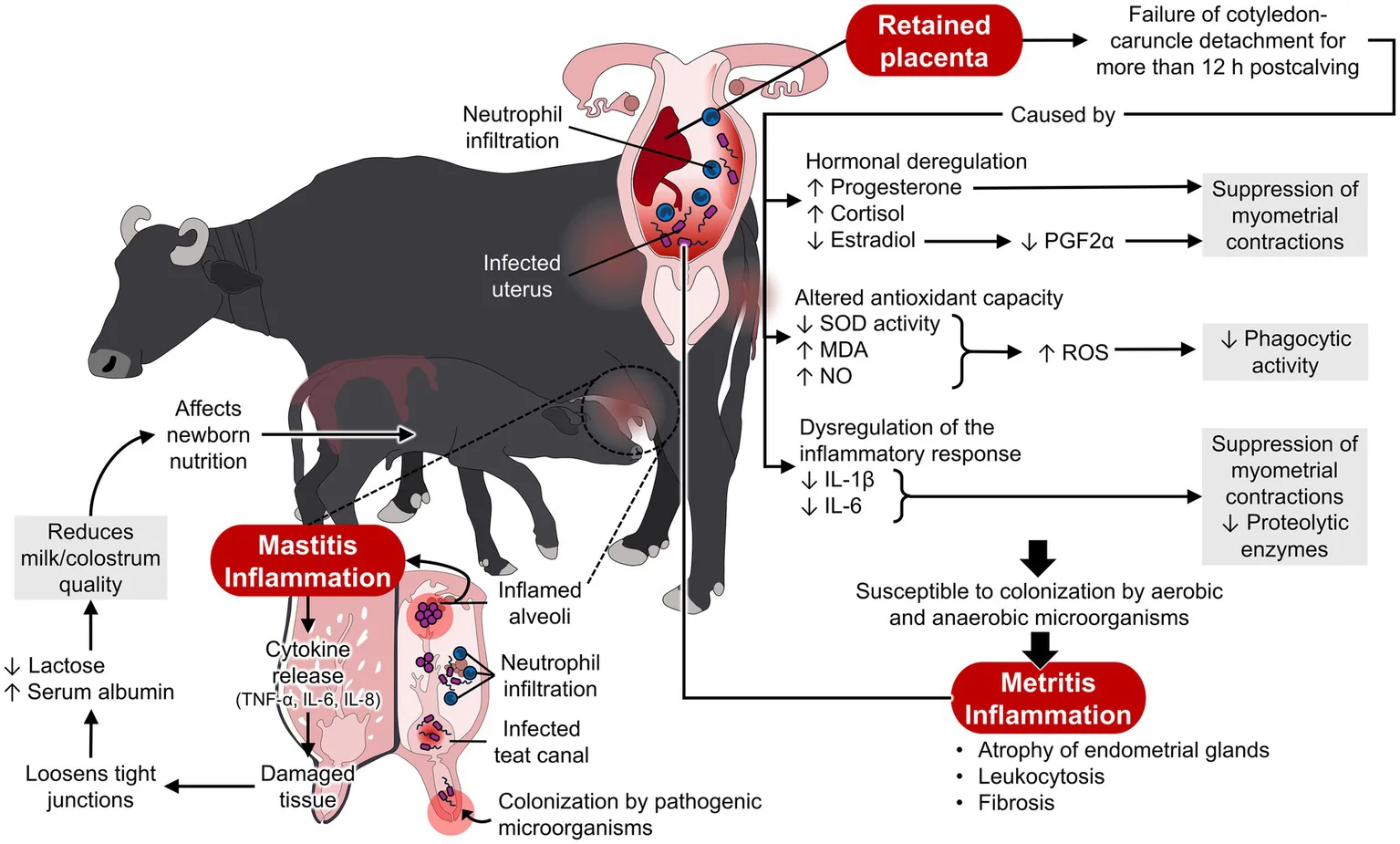
Post-calving disorders and their impact on maternal recovery and neonatal viability in water buffaloes. Retained placenta is a disorder associated with hormonal deregulation, altered antioxidant capacity, and a dysregulated inflammatory response. This disorder is related to metritis, a disorder that affects the buffalo dam’s health and other processes, such as uterine involution. Mastitis, another disease that can arise before or after calving, affects both the buffalo dam and the newborn by altering the quality and composition of colostrum/milk.

Metritis also leads to poor reproductive efficiency by delaying uterine involution, increasing days open, extending calving intervals, and affecting conception rates (138, 156, 159). For example, Rahawy (160) determined that the calving intervals of lactating Iraqi buffaloes with puerperal metritis were significantly longer (526.82 ± 7.42 days) than in healthy buffaloes (441.11 ± 6.36 days). Similarly, the open days were significantly longer in affected buffalo cows (203.4 ± 2.16 *vs*. 121.9 ± 5.17 days), which can also extend calving intervals. Figure 6 summarizes the main factors associated with post-calving pathologies in water buffaloes. (Figure 6).

#### 4.5.2 Mastitis

Mastitis is the inflammation of the parenchyma of the mammary gland, a condition that can be infectious or traumatic (Figure 6) (161, 162). Mastitis can be classified into subclinical and clinical forms. Clinical mastitis is characterized by physical, chemical, and bacteriological changes in the milk, together with changes in the mammary tissue (e.g., gland enlargement and asymmetry). Subclinical mastitis is characterized by normal gland and milk appearance, but an increase in somatic cell counts (SCC) (161). It is one of the most important diseases in dairy animals—including water buffaloes—due to the direct influence that it has on milk yield and milk chemical composition (161).

When compared with the incidence of mastitis in dairy cattle (*Bos taurus* and *Bos indicus*), water buffaloes have a low incidence of mammary gland pathologies, which is associated with the anatomical characteristics of the gland (163). The udder of buffalo cows is smaller than *Bos taurus* cattle. This has been reported by Abdullah et al. (163) in Nili-Ravi buffalo, in whom udder depth, width, and length (10.8 ± 1.6, 29.1 ± 4.1, and 64.2 ± 7.3 cm, respectively) were significantly smaller when compared to Holstein cattle (30.63, 50.52, and 50.78 cm, respectively). Furthermore, buffalo teats are longer (between 5 and 16 cm) with narrower channels (2.71 ± 0.10 cm) (164, 165), which might protect the udder from bacterial colonization.

The association between anatomical differences of the udder and the presence of subclinical mastitis has been evaluated by different authors. For example, Hussain et al. (166) reported that mastitic Nili-Ravi buffaloes had a pendulous udder with significantly higher udder depth (7.30 ± 1.00 in) when compared to healthy animals (5.55 ± 1.03 in). In addition, teat length was significantly lower in mastitic buffaloes (between 5.43 ± 0.15 and 5.68 ± 0.27 cm *vs*. 5.89 ± 0.37 and 6.46 ± 0.43 cm), and teat apex diameter was higher in all the quarters of mastitic buffaloes (between 1.04 ± 0.12 and 1.06 ± 0.09 cm *vs*. 0.79 ± 0.10 and 0.82 ± 0.12 cm). These findings suggest that mastitis is associated with teat parameters, as the entry of microorganisms through a short-length teat favors the infection of the mammary parenchyma. Similarly, in water buffaloes from Bangladesh, funnel-shaped teats (OR = 2.1) and asymmetrical udders (OR = 1.8) were associated with a higher prevalence of subclinical mastitis (167) due to the loose and rough skin of these teats, increasing the exposure to pathogens and the chance of injury during milking.

Studies have determined the etiology of subclinical mastitis in water buffalo, as Catozzi et al. (168) reported in milk samples from buffaloes with subclinical mastitis. *Acinetobacter* and *Pseudomonas* were the prevalent bacteria with 4.47 and 15.09%, respectively. Singha et al. (169) reported that the most common pathogen of mastitic buffaloes was non-aureus *staphylococci* (24.7%). In the same study, the occurrence of subclinical mastitis at the quarter and animal level was 42.5 and 81.6%, respectively, with an estimated SCC of 195,000 cells/mL milk. In Murrah buffaloes, *Staphylococcus*, *Streptococcus*, *Corynebacterium*, and *E. coli* were isolated (170). This is similar to what was found in Indian buffaloes, in whom the main microorganisms were *Staphylococci* (39%), *Streptococci* (31%), *Corynebacterium* (25%), and *E.coli* (5%) (171).

Among the predisposing factors for mastitis, poor management during milking is one of the main factors, either due to incomplete emptying of the udder or lack of hygiene at milking (162, 172, 173). In this sense, Singha et al. (167) determined the prevalence and risk factors associated with subclinical mastitis in water buffaloes from Bangladesh. A prevalence of 52% and a mean bulk milk SCC of 217,000 cells/mL of milk was found. The authors reported that most buffalo farms had poor hygiene practices with no antiseptic or teat-dipping techniques. Moreover, factors such as intensive rearing system (OR = 2.7) and milked by a single person (OR = 1.5) were related to subclinical mastitis.

Other factors have also been mentioned by Hussain et al. (166), who evaluated water buffaloes from mixed breed and Nili-Ravi breed with subclinical mastitis. A prevalence of 17.4% was recorded in mixed-breed buffaloes, while Nili Ravi buffaloes recorded 14.1%. It was also found that the right rear and front quarters were the most affected, with an incidence of 8.4 and 6.8%, respectively. A higher prevalence of subclinical mastitis (42.76%) was reported in lactating Bulgarian Murrah buffaloes (170), a prevalence of 53.66% for subclinical mastitis was reported in primiparous animals, while lactation lengths between 15 and 30 days were also associated with mastitis (OR = 2.4).

Acknowledging potential risk factors is key to managing mastitis in buffalo farms (166). For example, the implementation of hygienic measures might reduce the prevalence of mastitis, as investigated by Ng et al. (174), who implemented a livestock hygiene education program. Although the prevalence of mastitis was lower in trained households (39.4% *vs*. 60.4%), the difference was not statistically significant. However, results showed that trained households that washed buffalo teats after milking were less likely to have mastitis in their herd (OR = 0.25). Education and awareness about adequate management in the post-calving period can significantly reduce the prevalence of disorders such as mastitis and metritis, pathologies that reduce milk production, have consequences on the calf’s health, and represent significant economic losses for buffalo farmers (175).

Finally, one of the main consequences of mastitis is the changes in buffalo milk quality, which can affect calf health due to a lack of adequate nutrition. For example, Tripaldi et al. (176) reported that buffaloes with a total SCC above 200 × 10^3^/mL had significantly lower milk yields (4.73 ± 0.33 L/morning milking) and percentages of lactose (4.64 ± 0.05%) than healthy dams (5.79 ± 0.21 L/morning milking and 4.87 ± 0.03%, respectively). Similar findings were reported for Italian water buffaloes (177), and the authors mention that the altered milk quality is related to the increasing alveolar epithelium permeability, allowing lactose leaking due to the MG damage by inflammation, as reported in dairy cattle (178).

## 5 Future directions

Despite the substantial progress achieved in understanding the endocrine regulation of parturition, maternal behavior, neonatal adaptation, and postpartum physiology in water buffaloes, important knowledge gaps remain. Future research should further elucidate the neuroendocrine and sensory mechanisms underlying mother–offspring bonding and determine how environmental conditions, production systems, and maternal experience influence the establishment of this relationship and its consequences for neonatal survival, welfare, and long-term productivity (13, 54–56, 72, 179, 180). Beyond environmental, nutritional, and management factors, increasing evidence indicates that genetic variation also contributes to reproductive performance and disease susceptibility. Despite their generally low-to-moderate heritability, recent advances in genome-wide association studies (GWAS), transcriptomics, and other omics approaches have revealed genomic regions and biological pathways associated with fertility, calving interval, age at first calving, and reproductive efficiency (181–184). In Indian Murrah buffaloes, Vohra et al. (185) performed a GWAS using 38,560 SNPs to evaluate fertility-related traits, including age at sexual maturity, postpartum breeding interval, and breeding efficiency. Despite the absence of genome-wide significant SNPs, the highest-ranking loci were located near genes such as *APC, PDE11A, GRID2, IGF2BP3,* and *EDIL3*, providing promising targets for future validation. Complementing these findings, whole-blood transcriptome profiling in Murrah buffaloes revealed DEGs, SNPs, and SSRs associated with age at first calving and postpartum cyclicity, including *FKBP4* and *CACNA2D1*, together with genes involved in cell-cycle progression, hormonal and calcium signaling, and immune regulation (186). Taken together, these genomic and transcriptomic findings provide a foundation for biomarker-assisted selection strategies when integrated with physiological and environmental phenotypes. Despite these advances, their predictive value requires validation in independent populations under diverse production systems. Ultimately, integrating physiological, genomic, and multi-omics information represents a promising strategy for improving our understanding of reproductive adaptation and supporting genetic improvement programs in water buffaloes. Another major research priority is the implementation of precision livestock farming technologies capable of integrating behavioral, physiological, and environmental indicators for real-time monitoring of the periparturient period. The use of wearable sensors, automated behavioral analysis, computer vision, and artificial intelligence may facilitate earlier prediction of calving, timely detection of dystocia, and objective assessment of maternal and neonatal welfare, ultimately supporting evidence-based management strategies (2, 3, 38). Further studies should also focus on improving the understanding of colostrogenesis, passive transfer of immunity, and neonatal adaptation. In particular, research should clarify how maternal endocrine status, nutrition, mammary physiology, and colostrum quality interact to influence immune competence and early-life health, thereby contributing to improved survival and productive performance of buffalo calves (21, 97, 110, 122). Finally, future advances in perinatal physiology should increasingly be translated into practical reproductive management and preventive veterinary medicine. Optimizing reproductive programs, including fixed-time artificial insemination protocols, together with improving the prevention and clinical management of reproductive disorders such as uterine torsion and ovarian hypofunction, will contribute to enhancing reproductive efficiency, animal welfare, and the sustainability of buffalo production systems. Moreover, integrating physiological, behavioral, clinical, and genomic approaches will facilitate the development of evidence-based management strategies for buffalo production (182, 184, 187–189).

## 6 Conclusion

A comprehensive understanding of the endocrine, physiological, and behavioral dynamics across the pre-calving, calving, and post-calving period of water buffaloes (*Bubalus bubalis*) provides a solid scientific foundation for optimizing reproductive management, improving maternal health, and enhancing neonatal viability in buffalo production systems. The peripartum hormonal cascade—characterized by the progressive decline in progesterone, the rise in estradiol-17β, and the coordinated release of prostaglandin F2α and oxytocin—constitutes an integrated biological signal that orchestrates parturition onset, behavioral adaptations, and the functional activation of the mammary gland. Non-invasive behavioral indicators—including tail lifting, postural changes, sacrosciatic ligament relaxation, and decreased rumination time—offer practical tools for the early identification of calving onset and timely detection of dystocia in both intensive and extensive buffalo systems.

The dam–calf bond is a time-sensitive neuroendocrine process requiring uninterrupted physical contact within the first 6 h post-calving. The oxytocinergic system is central to this process, facilitating multisensory imprinting and selective maternal care. Parity significantly modulates bonding efficiency, with primiparous dams exhibiting greater neophobia, longer latencies to successful suckling, and higher rates of calf rejection compared with multiparous animals. Ensuring uninterrupted dam–calf contact during the sensitive period is therefore a management priority with direct consequences for the establishment of maternal behavior and successful passive immune transfer.

Colostrogenesis must be consolidated before the onset of copious milk secretion; once lactogenesis is fully triggered, Ig transport via the neonatal Fc receptor is progressively downregulated. The predominantly alveolar mammary morphology of water buffalo makes the milk ejection reflex essential for effective colostrum transfer to the neonate. Evidence reviewed in this article highlights the importance of timely colostrum intake and adequate colostrum quality to ensure successful passive immunity transfer in buffalo calves.

During the post-calving period, placental retention, metritis, and mastitis represent the main pathological threats to both the dam’s reproductive recovery and neonatal welfare. Peripartum hormonal imbalances—particularly estradiol deficiency and elevated progesterone and cortisol—contribute to oxidative and metabolic stress associated with placental retention and related postpartum complications. Mastitis, although less prevalent in buffaloes than in dairy cattle, compromises colostrum and milk quality and represents an underdiagnosed risk factor for neonatal morbidity that merits greater attention in perinatal management protocols.

From a practical perspective, the evidence reviewed in this article supports the implementation of integrated monitoring programs during the transition period in buffalo herds. For dams, routine assessment of body condition score, metabolic biomarkers (e.g., NEFA and BHBA), mineral status, and indicators of oxidative stress may assist in identifying animals at increased risk of negative energy balance, placental retention, uterine disorders, and reduced reproductive performance. For calves, monitoring colostrum quality and the success of passive immunity transfer provides valuable information regarding neonatal health and disease susceptibility. Together, these management strategies may contribute to improved maternal health, neonatal survival, welfare, and productive efficiency in buffalo production systems.

Several research gaps remain in bubaline perinatology. Species-specific studies on auditory and visual recognition during dam–calf bonding are largely absent, with most available data extrapolated from cattle and sheep. Standardized protocols for assessing passive immunity transfer and its failure in buffalo herds under diverse production systems—including tropical dual-purpose systems in Latin America and South Asia—are urgently needed. Validated automated monitoring tools adapted to the behavioral and physiological traits of *Bubalus bubalis* represent a priority for improving perinatal outcomes at scale. Integrating the mechanistic knowledge reviewed here into evidence-based perinatal management protocols will contribute to improved animal welfare, reduced neonatal mortality, and greater productive and reproductive efficiency in buffalo farming systems worldwide.

## References

[1] Mohammad DRI Abdel-Rahman MAM. A comparative study on behavioral, physiological, and adrenal changes in buffaloes during the first stage of labor with normal and difficult parturition. J Vet Behav. (2013) 8:46–50. doi: 10.1016/j.jveb.2012.04.005

[2] Teja A Jeyakumar S Rao KA Kumaresan A Ramesha KP Narayanan K . Pre- and peri parturient behaviour as an indicator of onset of the calving process in Murrah buffalo (*Bubalus bubalis*). Appl Anim Behav Sci. (2023) 263:105936. doi: 10.1016/j.applanim.2023.105936

[3] Lanzoni L Chincarini M Giammarco M Fusaro I Iannotta M Podaliri M . Changes in the behaviour before normal calving to predict its onset in Mediterranean buffaloes heifers. Appl Anim Behav Sci. (2022) 254:105721. doi: 10.1016/J.APPLANIM.2022.105721

[4] Mota-Rojas D Napolitano F Lanzoni L Pereira A Abd Ei-Aziz A Orihuela A . "Chapter 16. Welfare of female water buffaloes during calving and milking: physiological and behavioral aspects". In: Mota-Rojas D Orihuela A Napolitano F Baghieri A Ghezzi M Strappini A , editors. The Water Buffalo in the Americas: Behavior and Productivity. (In Spanish). México: BM Editores (2024). p. 441–74.

[5] Purohit GN Barolia Y Shekhar C Kumar P. Maternal dystocia in cows and buffaloes: A review. Open J Anim Sci. (2011) 1:41–53. doi: 10.4236/ojas.2011.12006

[6] Napolitano F Pacelli C Grasso F Braghieri A De Rosa G. The behaviour and welfare of buffaloes (*Bubalus bubalis*) in modern dairy enterprises. Animal. (2013) 7:1704–13. doi: 10.1017/S1751731113001109, 23803231

[7] Kumar P Sharma A Vasishta N Khan S Barman P. Dystocia due to relative oversized fetus and fetal maldisposition in a Buffalo. Vet World. (2011) 4:569. doi: 10.5455/vetworld.2011.569-570, 31622318

[8] Srinivas M Sreenu M Rani NL Naidu KS Prasad VD. Studies on dystocia in graded Murrah buffaloes: a retrospective study. Buffalo Bull. (2007) 26:40–5.

[9] Mota-Rojas D Orihuela A Strappini A Villanueva-García D Napolitano F Mora-Medina P . Consumption of maternal placenta in humans and nonhuman mammals: beneficial and adverse effects. Animals. (2020) 10:2398. doi: 10.3390/ani10122398, 33333890 PMC7765311

[10] González-Lozano M Mota-Rojas D Orihuela A Martínez-Burnes J Di Francia A Braghieri A . Review: behavioral, physiological, and reproductive performance of buffalo cows during eutocic and dystocic parturitions. Appl Anim Sci. (2020) 36:407–22. doi: 10.15232/AAS.2019-01946

[11] Hudson SJ Mullord MM. Investigations of maternal bonding in dairy cattle. Appl Anim Ethol. (1977) 3:271–6. doi: 10.1016/0304-3762(77)90008-6

[12] Bordi A De Rosa G Napolitano F Litterio M Marino V Rubino R. Postpartum development of the mother-young relationship in goats. Appl Anim Behav Sci. (1994) 42:145–52. doi: 10.1016/0168-1591(94)90154-6

[13] Mota-Rojas D Bienboire-Frosini C Marcet-Rius M Domínguez-Oliva A Mora-Medina P Lezama-García K . Mother-young bond in non-human mammals: neonatal communication pathways and neurobiological basis. Front Psychol. (2022) 13:1064444. doi: 10.3389/fpsyg.2022.1064444 36524176 PMC9744768

[14] Veissier I Caré S Pomiès D. Suckling, weaning, and the development of oral behaviours in dairy calves. Appl Anim Behav Sci. (2013) 147:11–8. doi: 10.1016/j.applanim.2013.05.002

[15] Mora-Medina P Napolitano F Mota-Rojas D Berdugo-Gutiérrez J Ruiz-Buitrago J Guerrero-Legarreta I. Imprinting, sucking and Allosucking behaviors in Buffalo calves. J Buffalo Sci. (2018) 7:49–57. doi: 10.6000/1927-520X.2018.07.03.3

[16] Agrawal S Kumar T Chaudhary R Lionel AP Kumar S. Assaying the concentration of immunoglobulin g in colostrum from females postpartum and serum from neonatal calves of buffaloes (*Bubalus Bubalis*). Buffalo Bull. (2022) 41:493–509. doi: 10.56825/bufbu.2022.4134858

[17] Mota-Rojas D Napolitano F Chay-Canul A Ghezzi M Braghieri A Domínguez-Oliva A . Anatomy and physiology of water buffalo mammary glands: an anatomofunctional comparison with dairy cattle. Animals. (2024) 14:1066. doi: 10.3390/ani14071066, 38612305 PMC11011071

[18] Baumrucker CR Bruckmaier RM. Colostrogenesis: IgG1 transcytosis mechanisms. J Mammary Gland Biol Neoplasia. (2014) 19:103–17. doi: 10.1007/s10911-013-9313-5, 24474529

[19] Singh SV Singh AV Singh R Sharma S Shukla N Misra S . Sero-prevalence of bovine Johne’s disease in buffaloes and cattle population of North India using indigenous ELISA kit based on native *Mycobacterium avium* subspecies paratuberculosis ‘Bison type’ genotype of goat origin. Comp Immunol Microbiol Infect Dis. (2008) 31:419–33. doi: 10.1016/J.CIMID.2007.06.002, 17854892

[20] Singh A Ahuja SP Singh B. Individual variation in the composition of colostrum and absorption of colostral antibodies by the precolostral buffalo calf. J Dairy Sci. (1993) 76:1148–56. doi: 10.3168/jds.S0022-0302(93)77443-3, 8486842

[21] de Souza DC da Silva DG Fonseca LCC de Castro FL Monteiro BM Bernardes O . Passive immunity transfer in water buffaloes (*Bubalus bubalis*). Front Vet Sci. (2020) 7:1–8. doi: 10.3389/fvets.2020.00247, 32626726 PMC7313533

[22] Cruz-Monterrosa RG Ramírez-Bribiesca JE Crosby-Galván MM Crosby-Galván EM Mota-Rojas D Domínguez-Vara IA . Physicochemical composition and long-chain fatty acids in fresh and freeze-dried colostrum from cows and buffaloes. Front Vet Sci. (2026) 13:1776116. doi: 10.3389/fvets.2026.1776116, 41994260 PMC13079004

[23] Thomas CS Svennersten-Sjaunja K Bhosrekar MR Bruckmaier RM. Mammary cisternal size, cisternal milk and milk ejection in Murrah buffaloes. J Dairy Res. (2004) 71:162–8. doi: 10.1017/S0022029904000081, 15190943

[24] Essawi WM El-Raghi AA Ali F Nassan MA Neamat-Allah ANF Hassan MAE. The Association of the Potential Risk Factors and Nutrition Elements with abortion and calving rates of Egyptian buffaloes (*Bubalus bubalis*). Animals. (2021) 11:2043. doi: 10.3390/ani11072043, 34359171 PMC8300411

[25] Akdağ A Okuyucu İC Erdem H Kul E Ocak N. Some welfare assessment traits and quantitative-qualitative Milk parameters as affected by supplementary feeding at milking and parity in Anatolian Buffalo cows. Animals. (2024) 14:956. doi: 10.3390/ani14060956, 38540054 PMC10967426

[26] Elsayed DH Abdelrazek HMA El-Azzazi FE Ismail SAA Mahmoud YK. Hormonal and metabolic profiles related to placental retention with emphasis on oxidative stress and serotonin receptors in pluriparous buffaloes. Reprod Dom Anim. (2020) 55:469–78. doi: 10.1111/rda.13640, 31961013

[27] Azawi OI Omran SN Hadad JJ. Clinical, bacteriological, and histopathological study of toxic puerperal metritis in Iraqi Buffalo. J Dairy Sci. (2007) 90:4654–60. doi: 10.3168/jds.2007-0114, 17881686

[28] Dodamani M Mohteshamuddin K Awati SD Tandle MK Honnapagol S. Study on calving pattern in buffaloes. Vet World. (2010) 3:188–90.

[29] Modi M Chauhan RA Shukla S. Process of parturition in buffaloes. Indian J Anim Reprod. (2002) 23:141–3.

[30] Arora RC Pandey RS. Changes in peripheral plasma concentrations of progesterone, estradiol-17β, and luteinizing hormone during pregnancy and around parturition in the buffalo (*Bubalus bubalis*). Gen Comp Endocrinol. (1982) 48:403–10. doi: 10.1016/0016-6480(82)90153-8, 7152242

[31] Batra SK Pahwa GS Pandey RS. Hormonal milieu around parturition in buffaloes (*Bubalus bubalis*). Biol Reprod. (1982) 27:1055–61. doi: 10.1095/biolreprod27.5.1055, 6961942

[32] Kumar V Kumar P Mohan K Sarkar M Suresh KP Chauhan MS . Temporal changes in circulating levels of plasma interleukin-8 during peripartum period in Murrah buffaloes (*Bubalus bubalis*) under subtropical climate. Trop Anim Health Prod. (2011) 43:669–74. doi: 10.1007/s11250-010-9751-7, 21107908

[33] Singh IS Gupta A Nagarsekar A Cooper Z Manka C Hester L . Heat shock co-activates Interleukin-8 transcription. Am J Resp Cell Mol Biol. (2008) 39:235–42. doi: 10.1165/rcmb.2007-0294OC, 18367728 PMC2542457

[34] Kimura K Goff JP Kehrli ME Reinhardt TA. Decreased neutrophil function as a cause of retained placenta in dairy cattle. J Dairy Sci. (2002) 85:544–50. doi: 10.3168/jds.S0022-0302(02)74107-6, 11949858

[35] Deka R Nath KC Bhuyan M Bhuyan D Das GC Dutta L . Parturition behavior of swamp buffalo cows (*Bubalus bubalis*) under organized system of rearing. Biol Rhy Res. (2021) 52:444–53. doi: 10.1080/09291016.2019.1607220

[36] Singh R Khar S Chander S. Parturition in buffaloes. Indian J Anim Sci. (1994) 64:1028–33.

[37] Das G Kumar A Dangi S Kham F. "Parturition and puerperium in the buffalo". In: Purohit G, editor. Bubaline Theriogenology. Ithaca: International Veterinary Information Service (2013). p. 238–51.

[38] Rossi E Ferri N Crociati M Monaci M Stradaioli G Sylla L. Remote monitoring system as a tool for calving management in Mediterranean Buffalo heifers (*Bubalus bubalis*). Reprod Dom Anim. (2020) 55:1803–7. doi: 10.1111/rda.13805, 32780888

[39] Giaretta E Marliani G Postiglione G Magazzù G Pantò F Mari G . Calving time identified by the automatic detection of tail movements and rumination time, and observation of cow behavioural changes. Animal. (2021) 15:100071. doi: 10.1016/j.animal.2020.100071, 33516029

[40] Quddus RA Ahmad N Khalique A Bhatti JA. Evaluation of automated monitoring calving prediction in dairy buffaloes a new tool for calving management. Braz J Biol. (2022) 82:e257884. doi: 10.1590/1519-6984.257884, 35544790

[41] Mota-Rojas D Napolitano F Strappini A Orihuela A Ghezzi MD Hernández-Ávalos I . Pain at the slaughterhouse in ruminants with a focus on the neurobiology of sensitisation. Animals. (2021) 11:1085. doi: 10.3390/ani11041085, 33920244 PMC8068923

[42] Barraclough RAC Shaw DJ Boyce R Haskell MJ Macrae AI. The behavior of dairy cattle in late gestation: effects of parity and dystocia. J Dairy Sci. (2020) 103:714–22. doi: 10.3168/jds.2019-16500, 31629521

[43] Miedema HM Cockram MS Dwyer CM Macrae AI. Changes in the behaviour of dairy cows during the 24h before normal calving compared with behaviour during late pregnancy. Appl Anim Behav Sci. (2011) 131:8–14. doi: 10.1016/j.applanim.2011.01.012

[44] Burfeind O Suthar VS Voigtsberger R Bonk S Heuwieser W. Validity of prepartum changes in vaginal and rectal temperature to predict calving in dairy cows. J Dairy Sci. (2011) 94:5053–61. doi: 10.3168/jds.2011-4484, 21943756

[45] Ricci A Racioppi V Iotti B Bertero A Reed KF Pascottini OB . Assessment of the temperature cut-off point by a commercial intravaginal device to predict parturition in Piedmontese beef cows. Theriogenology. (2018) 113:27–33. doi: 10.1016/j.theriogenology.2018.02.009, 29452854

[46] Mota-Rojas D Titto CG Orihuela A Martínez-Burnes J Prado J Torres-Bernal F . Physiological and behavioral mechanisms of thermoregulation in mammals. Animals. (2021) 11:1733. doi: 10.3390/ANI11061733, 34200650 PMC8227286

[47] Bertoni A Mota-Rojas D Álvarez-Macias A Mora-Medina P Guerrero-Legarreta I Morales-Canela A . Scientific findings related to changes in vascular microcirculation using infrared thermography in the river buffalo. J Anim Behav Biometeorol. (2020) 8:288–97. doi: 10.31893/JABB.20038

[48] Mota-Rojas D Napolitano F Braghieri A Guerrero-Legarreta I Bertoni A Martínez-Burnes J . Thermal biology in river buffalo in the humid tropics: neurophysiological and behavioral responses assessed by infrared thermography. J Anim Behav Biometeorol. (2021) 9:e2103. doi: 10.31893/JABB.21003

[49] Mota-Rojas D Braghieri A Ghezzi M Ceriani MC Martínez-Burnes J Lendez PA . Strategies and mechanisms of thermal compensation in newborn water buffaloes. Animals. (2023) 13:2161. doi: 10.3390/ani13132161, 37443964 PMC10340076

[50] Mota-Rojas D Pereira AMF Wang D Martínez-Burnes J Ghezzi M Hernández-Avalos I . Clinical applications and factors involved in validating thermal windows used in infrared thermography in cattle and river buffalo to assess health and productivity. Animals. (2021) 11:2247. doi: 10.3390/ani11082247, 34438705 PMC8388381

[51] Mota-Rojas D Wang D Titto CG Prado G Carvajal-de la Fuente V Ghezzi M . Pathophysiology of fever and application of infrared thermography (IRT) in the detection of sick domestic animals: recent advances. Animals. (2021) 11:2316. doi: 10.3390/ani11082316, 34438772 PMC8388492

[52] Mota-Rojas D Wang D Titto CG Martínez-Burnes J Villanueva-García D Lezama K . Neonatal infrared thermography images in the hypothermic ruminant model: anatomical-morphological-physiological aspects and mechanisms for thermoregulation. Front Vet Sci. (2022) 9:963205. doi: 10.3389/fvets.2022.963205, 35990264 PMC9386124

[53] Napolitano F Bragaglio A Braghieri A El-Aziz AHA Cristiane G Villanueva-garcía D . The effect of birth weight and time of day on changes in the dermal microcirculation of water buffalo calves during the first days of life. Front. (2023) 10:1084092. doi: 10.3389/fvets.2023.1084092, 36925607 PMC10011160

[54] Mota-Rojas D Bienboire-Frosini C Orihuela A Domínguez-Oliva A Villanueva García D Mora-Medina P . Mother–offspring bonding after calving in water Buffalo and other ruminants: sensory pathways and neuroendocrine aspects. Animals. (2024) 14:2696. doi: 10.3390/ani14182696, 39335285 PMC11428873

[55] Mota-Rojas D Bragaglio A Braghieri A Napolitano F Domínguez-Oliva A Mora-Medina P . Dairy Buffalo behavior: calving, imprinting and Allosuckling. Animals. (2022) 12:2899. doi: 10.3390/ANI12212899, 36359022 PMC9658508

[56] Orihuela A Mota-Rojas D Strappini A Serrapica F Braghieri A Mora-Medina P . Neurophysiological mechanisms of cow–calf bonding in Buffalo and other farm animals. Animals. (2021) 11:1968. doi: 10.3390/ANI11071968, 34209286 PMC8300112

[57] Dubey P Singh RR Choudhary SS Verma KK Kumar A Gamit PM . Post parturient neonatal behaviour and their relationship with maternal behaviour score, parity and sex in Surti buffaloes. J Appl Anim Res. (2018) 46:360–4. doi: 10.1080/09712119.2017.1306533

[58] Lanzoni L Chincarini M Giammarco M Fusaro I Gloria A Contri A . Maternal and neonatal behaviour in italian mediterranean buffaloes. Animals. (2021) 11:1584. doi: 10.3390/ani11061584, 34071324 PMC8226658

[59] Rodríguez-González D Minervino AHH Orihuela A Bertoni A Morales-Canela DADA Álvarez-Macías A . Handling and physiological aspects of the dual-purpose water Buffalo production system in the Mexican humid tropics. Animals. (2022) 12:608. doi: 10.3390/ani12050608, 35268176 PMC8909038

[60] Sreedhar S Ranganadham M Mohan E. Calf mortality in indigenous buffaloes. Indian Vet J. (2010) 87:197–8. doi: 10.5555/20103080979

[61] Booth K Katz LS. Role of the Vomeronasal organ in neonatal offspring recognition in sheep. Biol Reprod. (2000) 63:953–8. doi: 10.1095/biolreprod63.3.953, 10952943

[62] Mesgarani N David SV Fritz JB Shamma SA. Mechanisms of noise robust representation of speech in primary auditory cortex. Proceed Nati AcaD Sci USA. (2014) 111:6792–7. doi: 10.1073/pnas.1318017111, 24753585 PMC4020083

[63] Acevedo-Rodriguez A Mani SK Handa RJ. Oxytocin and estrogen receptor β in the brain: an overview. Front Endocrinol. (2015) 6:160. doi: 10.3389/fendo.2015.00160 PMC4606117 26528239

[64] Nowak R Keller M Val-Laillet D Lévy F. Perinatal visceral events and brain mechanisms involved in the development of mother–young bonding in sheep. Horm Behav. (2007) 52:92–8. doi: 10.1016/J.YHBEH.2007.03.021, 17488646

[65] Nagasawa M Okabe S Mogi K Kikusui T. Oxytocin and mutual communication in mother-infant bonding. Front Human Neurosci. (2012) 6:31. doi: 10.3389/fnhum.2012.00031, 22375116 PMC3289392

[66] Poindron P Lévy F Krehbiel D. Genital, olfactory, and endocrine interactions in the development of maternal behaviour in the parturient ewe. Psychoneuro. (1988) 13:99–125. doi: 10.1016/0306-4530(88)90009-1, 3287420

[67] Nakayama S Nakamitsu D Aomori D Ohigashi S Nishino O Tose S. A novel method for parturition induction in cattle using vaginal wall stimulation. Theriogenol. (2025) 245:117522. doi: 10.1016/j.theriogenology.2025.117522, 40489933

[68] Peeters G de VN Houvenaghel A. Elimination of Ferguson reflex by section of the pelvic nerves in the lactating goat. J Endocrinol. (1971) 49:125–30. doi: 10.1677/joe.0.0490125, 5104448

[69] Kendrick KM Lévy F Keverne EB. Importance of vaginocervical stimulation for the formation of maternal bonding in primiparous and multiparous parturient ewes. Physiol Behav. (1991) 50:595–600. doi: 10.1016/0031-9384(91)90551-X, 1801015

[70] López-Arjona M Mainau E Navarro E Contreras-Aguilar MD Escribano D Mateo SV . Oxytocin in bovine saliva: validation of two assays and changes in parturition and at weaning. BMC Vet Res. (2021) 17:140. doi: 10.1186/S12917-021-02838-5, 33794896 PMC8017845

[71] Kendrick KM Keverne EB Baldwin BA Sharman DF. Cerebrospinal fluid levels of acetylcholinesterase, monoamines and oxytocin during labour, parturition, vaginocervical stimulation, lamb separation and suckling in sheep. Neuroendocrinol. (1986) 44:149–56. doi: 10.1159/000124638, 3796790

[72] Nowak R Keller M Lévy F. Mother-young relationships in sheep: A model for a multidisciplinary approach of the study of attachment in mammals. J Neuroendocrinol. (2011) 23:1042–53. doi: 10.1111/J.1365-2826.2011.02205.X, 21827554

[73] Napolitano F Orihuela A Strappini AC Guerrero-Legarreta I Rodríguez-González D Barile VL . "Chapter 11. Comprehensive water Buffalo production: basic aspects of obstetrics, imprinting, lactation, meat science, and regulations". In: Mota-Rojas D Orihuela A Napolitano F Braghieri A Ghezzi M Strappini AC , editors. The Water Buffalo in the Americas: Behavior and Productivity. (In Spanish). Mexico City: B.M. Editores (2024). p. 309–46.

[74] Mota-Rojas D Bienboire-Frosini C Bettencourt AF Villanueva-García D Domínguez-Oliva A Álvarez-Macías A . Failure in the mother-young communication in domestic mammals: endocrine and behavioral aspects. Front Vet Sci. (2025) 12:1589916. doi: 10.3389/fvets.2025.1589916, 40510376 PMC12158967

[75] Coppola DM Millar LC. Stimulus access to the accessory olfactory system in the prenatal and perinatal rat. Neurosci. (1994) 60:463–8. doi: 10.1016/0306-4522(94)90257-7, 8072692

[76] Insley SJ. Mother–offspring vocal recognition in northern fur seals is mutual but asymmetrical. Anim Behav. (2001) 61:129–37. doi: 10.1006/anbe.2000.1569, 11170703

[77] Baldwin BA Shillito EE. The effects of ablation of the olfactory bulbs on parturition and maternal behaviour in soay sheep. Anim Behav. (1974) 22:220–3. doi: 10.1016/S0003-3472(74)80072-2, 4836744

[78] Lévy F Keller M Poindron P. Olfactory regulation of maternal behavior in mammals. Horm Behav. (2004) 46:284–302. doi: 10.1016/J.YHBEH.2004.02.005, 15325229

[79] Tulloch DG. The water Buffalo, *Bubalus Bubalis*, in Australia: reproductive and parent-offspring behaviour. Wild Res. (1979) 6:265–87. doi: 10.1071/WR9790265

[80] Whalin L Weary DM von Keyserlingk MAG. Understanding Behavioural development of calves in natural settings to inform calf management. Animals. (2021) 11:2446. doi: 10.3390/ani11082446, 34438903 PMC8388734

[81] de FM Oliveira A Quirino CR Bastos R. Effect of nursing behaviour, sex of the calf, and parity order on milk production of buffaloes. Rev Colomb Ciencias Pecu. (2017) 30:30–8. doi: 10.17533/udea.rccp.v30n1a04

[82] Edwards SA Broom DM. Behavioural interactions of dairy cows with their newborn calves and the effects of parity. Anim Behav. (1982) 30:525–35. doi: 10.1016/S0003-3472(82)80065-1

[83] Lenner Á Papp ZL Komlósi I. Analysis of sounds made by Bos taurus and *Bubalus bubalis* dams to their calves. Front Vet Sci. (2025) 12:1549100. doi: 10.3389/fvets.2025.1549100, 40171411 PMC11960746

[84] Padilla de la Torre M Briefer EF Reader T McElligott AG. Acoustic analysis of cattle (*Bos taurus*) mother–offspring contact calls from a source–filter theory perspective. *Appl Anim*. Behav Sci. (2015) 163:58–68. doi: 10.1016/j.applanim.2014.11.017, 38826717

[85] Alexander G Shillito Walser EE. Visual discrimination between ewes by lambs. Appl Anim Ethol. (1978) 4:81–5. doi: 10.1016/0304-3762(78)90096-2

[86] Hudson R. Olfactory imprinting. Curr Opin Neurobiol. (1993) 3:548–52. doi: 10.1016/0959-4388(93)90054-3, 8219720

[87] Saqib MN Qureshi MS Suhail SM Khan RU Bozzo G Ceci E . Association among metabolic status, oxidative stress, milk yield, body condition score and reproductive cyclicity in dairy buffaloes. Reprod Dom Anim. (2022) 57:498–504. doi: 10.1111/RDA.14086, 35066924 PMC9306642

[88] Napolitano F Braghieri A Bragaglio A Rodríguez-González D Mora-Medina P Ghezzi MD . Neurophysiology of Milk ejection and Prestimulation in dairy buffaloes. Animals. (2022) 12:2649. doi: 10.3390/ani12192649, 36230390 PMC9559521

[89] Lotito D Pacifico E Matuozzo S Musco N Iommelli P Tudisco R . Colostrum management for buffalo calves: a review. Vet Sci. (2023) 10:1–12. doi: 10.3390/vetsci10050358 PMC10222353 37235441

[90] Barrington GM McFadden TB Huyler MT Besser TE. Regulation of colostrogenesis in cattle. Livest Prod Sci. (2001) 70:95–104. doi: 10.1016/S0301-6226(01)00201-9

[91] Coroian A Erler S Matea CT Mireșan V Răducu C Bele C . Seasonal changes of buffalo colostrum: physicochemical parameters, fatty acids andcholesterol variation. Chem Cent J. (2013) 7:40. doi: 10.1186/1752-153X-7-40, 23442377 PMC3604957

[92] Brandon M Watson D Lascelles A. The mechanism of transfer of immunoglobulin into mammary secretion of cows. Aust J Exp Biol Med Sci. (1971) 49:613–23. doi: 10.1038/icb.1971.67, 5153736

[93] Abdelsattar MM Rashwan AK Younes HA Abdel-Hamid M Romeih E Mehanni A-HE . An updated and comprehensive review on the composition and preservation strategies of bovine colostrum and its contributions to animal health. Anim Feed Sci Technol. (2022) 291:115379. doi: 10.1016/j.anifeedsci.2022.115379

[94] Puppel K Gołębiewski M Grodkowski G Slósarz J Kunowska-Slósarz M Solarczyk P . Composition and factors affecting quality of bovine colostrum: a review. Animals. (2019) 9:1070. doi: 10.3390/ani9121070, 31810335 PMC6940821

[95] Stark A Wellnitz O Dechow C Bruckmaier R Baumrucker C. Colostrogenesis during an induced lactation in dairy cattle. J Anim Physiol Anim Nutrit. (2015) 99:356–66. doi: 10.1111/jpn.12205, 24828984

[96] Castro N Capote J Bruckmaier RM Argüello A. Management effects on colostrogenesis in small ruminants: a review. J Appl Anim Res. (2011) 39:85–93. doi: 10.1080/09712119.2011.581625

[97] Bigler NA Gross JJ Baumrucker CR Bruckmaier RM. Endocrine changes during the peripartal period related to colostrogenesis in mammalian species. J Anim Sci. (2023) 101:skad146. doi: 10.1093/jas/skad146, 37158662 PMC10237234

[98] Convey EM. Serum hormone concentration in ruminants during mammary growth, Lactogenesis, and lactation: A review. J Dairy Sci. (1974) 57:905–17. doi: 10.3168/jds.S0022-0302(74)84986-6, 4369168

[99] Stumpf MT Fischer V Daltro DS Alfonzo EPM Kolling GJ da Silva MVGB . Mammary gland cell’s tight junction permeability from dairy cows producing stable or unstable milk in the ethanol test. Int J Biometeorol. (2020) 64:1981–3. doi: 10.1007/s00484-020-01967-0, 32691150

[100] Kessler EC Wall SK Hernandez LL Gross JJ Bruckmaier RM. Short communication: mammary gland tight junction permeability after parturition is greater in dairy cows with elevated circulating serotonin concentrations. J Dairy Sci. (2019) 102:1768–74. doi: 10.3168/jds.2018-15543, 30580948

[101] Fleet IR Peaker M. Mammary function and its control at the cessation of lactation in the goat. J Physiol. (1978) 279:491–507. doi: 10.1113/jphysiol.1978.sp012358, 671360 PMC1282629

[102] Zhao X Ponchon B Lanctôt S Lacasse P. Invited review: accelerating mammary gland involution after drying-off in dairy cattle. J Dairy Sci. (2019) 102:6701–17. doi: 10.3168/jds.2019-16377, 31202662

[103] Chaurasia D Dalvi RS Banubakode SB Netty S Kumar DS Mandavi S. Histoarchitectural study of mammary alveoli on lactation, involution and pregnant stage in Murrah buffalo. Buffalo Bull. (2022) 41:19–2. doi: 10.56825/bufbu.2022.4112497

[104] Morgan G Wooding FBP. A freeze-fracture study of tight junction structure in sheep mammary gland epithelium during pregnancy and lactation. J Dairy Res. (1982) 49:1–11. doi: 10.1017/S002202990002207X, 7076943

[105] Claude P Goodenough DA. Fracture faces of zonulae Occludentes from “tight” and “leaky” epithelia. J Cell Biol. (1973) 58:390–400. doi: 10.1083/jcb.58.2.390, 4199658 PMC2109050

[106] Abdelrahman M Liu G Al-Saeed FA Liu Y Hou F Yang H . Deciphering the colostral-immunity transfer: from mammary gland to neonates small intestine. Vet Res Commun. (2025) 49:72. doi: 10.1007/S11259-025-10646-7, 39798032

[107] Agrawal S Kumar S Chaudhary R Chauhan A Kumar A Sivamani B. Allele mining in gene encoding fc fragment of IgG receptor, transporter, alpha (FCGRT) and association of nucleotide variants with passive transfer of immunity in neonatal buffalo calves. Mol Biol Rep. (2023) 50:3429–38. doi: 10.1007/S11033-023-08267-X, 36757548

[108] Stark A Vachkova E Wellnitz O Bruckmaier R Baumrucker C. Colostrogenesis: candidate genes for IgG1 transcytosis mechanisms in primary bovine mammary epithelial cells. J Anim Physiol Anim Nutr (Berl). (2013) 97:1114–24. doi: 10.1111/jpn.12021, 23279563

[109] Korhonen H Marnila P Gill HS. Milk immunoglobulins and complement factors. Br J Nutr. (2000) 84:75–80. doi: 10.1017/S0007114500002282, 11242450

[110] Giammarco M Chincarini M Fusaro I Manetta AC Contri A Gloria A . Evaluation of brix Refractometry to estimate immunoglobulin G content in Buffalo colostrum and neonatal calf serum. Animals. (2021) 11:2616. doi: 10.3390/ani11092616, 34573582 PMC8464908

[111] Abd EI-Fattah AM Abd Rabo FH EL-Dieb SM El-Kashef HA. Changes in composition of colostrum of Egyptian buffaloes and Holstein cows. BMC Vet Res. (2012) 8:1, 19–7. doi: 10.1186/1746-6148-8-19, 22390895 PMC3344693

[112] Souza DC Silva DG Rocha TG Monteiro BM Pereira GT Fiori LC . Serum biochemical profile of neonatal buffalo calves. Arq Bras Med Vet Zootec. (2019) 71:187–96. doi: 10.1590/1678-4162-10176

[113] Godden SM Lombard JE Woolums AR. Colostrum Management for Dairy Calves. Vet Clin North Am: Food Animal Pract. (2019) 35:535–56. doi: 10.1016/j.cvfa.2019.07.005, 31590901 PMC7125574

[114] Mayasari N de Vries RG Nieuwland MGB Remmelink GJ Parmentier HK Kemp B . Effect of maternal dry period length on colostrum immunoglobulin content and on natural and specific antibody titers in calves. J Dairy Sci. (2015) 98:3969–79. doi: 10.3168/jds.2014-8753, 25828658

[115] Van Hese I Goossens K Vandaele L Ampe B Haegeman A Opsomer G. The effect of maternal supply of rumen-protected protein to Holstein Friesian cows during the dry period on the transfer of passive immunity and colostral microbial composition. J Dairy Sci. (2023) 106:8723–45. doi: 10.3168/JDS.2023-23266, 37678775

[116] Sikka P Lal D. Studies on vitamin mineral interactions in relation to passive transfer of immunoglobulins in Buffalo calves. Asian-Australasian J Anim Sci. (2006) 19:825–30. doi: 10.5713/ajas.2006.825

[117] Sikka P Lall D Arora U Sethi R. Growth and passive immunity in response to micronutrient supplementation in new-born calves of Murrah buffaloes given fat soluble vitamins during late pregnancy. Livest Prod Sci. (2002) 75:301–11. doi: 10.1016/S0301-6226(01)00325-6

[118] Sikka P Lal D Khanna S Tomer AKS Sethi RK. Studies on colostrum minerals and their transfer to suckling calves in buffaloes. Indian J Anim Sci. (2011) 76:480–3.

[119] Karthik D Gangaraju G Reddy YPK Suresh J Praneeth D. Effect of prepartum dry period on the postpartum body weight and milk yield of dairy buffaloes. Int J Curr Res. (2020) 9:2875–82.

[120] Nagarjuna Reddy A Seshiah CV Sudhakar K Srinivasa Kumar D Ravi Kanth Reddy P. Effects of shortened dry period on the physical indicators of energy reserves mobilization in high yielding Murrah buffaloes. Indian J Anim Res. (2018) 52:1656–60. doi: 10.18805/ijar.B-3406

[121] Reddy AN Seshiah CV Sudhakar K Kumar DS Reddy PRK. Shortened dry period in dairy buffaloes: influence on milk yield, milk components and reproductive performance. Indian J Anim Res. (2019) 53:119–23. doi: 10.18805/ijar.B-3457

[122] Lotito D Pacifico E Matuozzo S Musco N Iommelli P Zicarelli F . Colostrum composition, characteristics and Management for Buffalo Calves: A review. Vet Sci. (2023) 10:358. doi: 10.3390/VETSCI10050358, 37235441 PMC10222353

[123] Weaver DM Tyler JW VanMetre DC Hostetler DE Barrington GM. Passive transfer of Colostral immunoglobulins in calves. J Vet Int Med. (2000) 14:569–77. doi: 10.1111/j.1939-1676.2000.tb02278.x, 11110376

[124] Dang AK Kapila S Purohit M Singh C. Changes in colostrum of Murrah buffaloes after calving. Trop Anim Health Produc. (2009) 41:1213–7. doi: 10.1007/s11250-008-9302-7, 19105042

[125] Sikka P Sethi RK. Blood serum immunoglobulin pattern in Murrah buffalo calves. Int J Anim Sci. (1997) 12:155–7.

[126] Mastellone V Massimini G Pero ME Lombardi P Britti D Avallone L. Passive transfer status and growth performance in newborn buffalo calves allowed to nurse the dam. Ital J Anim Sci. (2007) 6:1245–8. doi: 10.4081/ijas.2007.s2.1245

[127] Bush LJ Staley TE. Absorption of Colostral immunoglobulins in newborn calves. J Dairy Sci. (1980) 63:672–80. doi: 10.3168/jds.S0022-0302(80)82989-4, 6991559

[128] Staley TE Bush LJ. Receptor mechanisms of the neonatal intestine and their relationship to immunoglobulin absorption and disease. J Dairy Sci. (1985) 68:184–205. doi: 10.3168/jds.S0022-0302(85)80812-2, 3884680

[129] Zaman T Khan A Akhtar MZ. Some of the risk factors of Nili-Ravi buffalo (*Bubalus bubalis*) neonatal calf mortality in Pakistan. Pak Vet J. (2006) 26:121–5.

[130] Okuyucu İC Erdem H. The effects of colostrum nutritional components, colostrum and serum bioactive substances on the morbidity, mortality and growth performance of water buffalo (*Bubalus bubalis*) calves until weaning. Trop Anim Health Prod. (2025) 57:350. doi: 10.1007/s11250-025-04605-2, 40767983

[131] Costa A De Marchi M Visentin G Campagna MC Borghese A Boselli C. The effect of pre-milking stimulation on teat morphological parameters and Milk traits in the Italian water Buffalo. Front Vet Sci. (2020) 7:572422. doi: 10.3389/fvets.2020.572422, 33364254 PMC7752857

[132] Tsingotjidou AS Papadopoulos GC. Anatomic organization of the ascending branch of the milk-ejection reflex in sheep: primary afferent neurons. J Comp Neurol. (2003) 460:66–79. doi: 10.1002/cne.10641, 12687697

[133] Akers RM Lefcourt AM. Milking- and suckling-induced secretion of oxytocin and prolactin in parturient dairy cows. Horm Behav. (1982) 16:87–93. doi: 10.1016/0018-506X(82)90009-5, 7068129

[134] Tancin V Kraetzl W-D Schams D Bruckmaier RM. The effects of conditioning to suckling, milking and of calf presence on the release of oxytocin in dairy cows. Appl Anim Behav Sci. (2001) 72:235–46. doi: 10.1016/S0168-1591(01)00113-7, 11311417

[135] Bruckmaier RM. Normal and disturbed milk ejection in dairy cows. Domes Anim Endocrinol. (2005) 29:268–73. doi: 10.1016/j.domaniend.2005.02.023, 15998500

[136] Tsingotjidou AS Papadopoulos GC. The Milk-ejection reflex in the sheep: an anatomical study on the afferent pathway. Anat Histol Embryol. (2008) 37:245–50. doi: 10.1111/j.1439-0264.2007.00833.x, 18205887

[137] Kosmidis EK Tsingotjidou AS Iliadis L Batzios C Papadopoulos GC. A sparsely connected network to model the relay stations of the sheep milk ejection reflex. Neurocomputing. (2009) 73:80–6. doi: 10.1016/j.neucom.2008.08.022

[138] Azawi O Omran S Hadad J. A study on postpartum metritis in Iraqi Buffalo cows: bacterial causes and treatment. Reprod Domes Anim. (2008) 43:556–65. doi: 10.1111/j.1439-0531.2007.00952.x, 18363608

[139] Lisuzzo A Mazzotta E Cappelli G Martucciello A Monteiro BM Sales JNS . Biochemical profile differences during the transition period based on different levels of non-esterified fatty acids at 7 weeks before parturition in Mediterranean Italian dairy buffaloes (*Bubalus bubalis*). Front Vet Sci. (2024) 11:1404041. doi: 10.3389/fvets.2024.1404041, 39015111 PMC11250106

[140] Ahmed W Abd El Hameed AR ElKhadrawy H Hanafi EM. Investigations on retained placenta in Egyptian buffaloes. Glob Vet. (2009) 3:120–4.

[141] Hassan M Nasreldin N El-Zeftawy M. Serum biochemical investigations on retained placenta in Egyptian buffaloes. Advan Anim Vet Sci. (2019) 8:67–76. doi: 10.17582/journal.aavs/2020/8.1.67.76

[142] Patel RV Parmar SC. Retention of fetal membranes and its clinical perspective in bovines. Sch J Agric Vet Sci. (2016) 3:111–6. doi: 10.36347/sjavs.2016.v03i02.008

[143] Gupta A Pandit R Jogi S Agarwal R. Retention of placenta in relation to parity season and sex of calf in Murrah buffaloes. Buffalo Bull. (1999) 18:5–7.

[144] Indurkar S Tiwari RP Dubey M Khan JR Mishra GK. Biochemical and hormonal profiles in buffaloes with retained fetal membranes. Buffalo Bull. (2019) 38:35–9.

[145] Gohar MA Elmetwally MA Montaser A Zaabel SM. Effect of Oxytetracycline treatment on postpartum reproductive performance in dairy Buffalo-cows with retained placenta in Egypt. J Vet Healthc. (2018) 1:45–53. doi: 10.14302/issn.2575-1212.jvhc-18-2146

[146] Ganaie BA Japheth KP Ali M Lone SA Mir SH Malik TA. An insight into the pathophysiology, preventive and treatment strategies of retained fetal membranes in bovines A review. J Anim Health Prod. (2018) 6:62–72. doi: 10.17582/journal.jahp/2018/6.2.62.72, 42511068

[147] Naief A-SA Al Khazraji AAH Al-Anbari NN Al-Saeidy HH Hasan HA. The incidence of postpartum metritis and retained placenta in cattle and Buffalo cows in some villages around Baghdad. J Buffalo Sci. (2015) 4:59–63. doi: 10.6000/1927-520x.2015.04.03.1

[148] Atallah S Moustafa S. Relation between oxidative stress and retained placenta in buffaloes. Assiut Vet Med J. (2006) 52:298–311. doi: 10.21608/avmj.2006.177408, 31961013

[149] Shynlova O Nadeem L Lye S. Progesterone control of myometrial contractility. J Steroid Biochem Mol Biol. (2023) 234:106397. doi: 10.1016/j.jsbmb.2023.106397, 37683774

[150] Uvnäs-Moberg K. The physiology and pharmacology of oxytocin in labor and in the peripartum period. Am J Obst Gynecol. (2024) 230:S740–58. doi: 10.1016/j.ajog.2023.04.011, 38462255

[151] Simões J Medeiros I. "Retained placenta in cattle". In: Simoes J, editor. Encyclopedia of Livestock Medicine for Large Animal and Poultry Production. Cham: Springer Nature Switzerland (2026). p. 1141–8.

[152] Andrei S Rafa H Oroian I Cozma OM Morohoschi AG Dumitraș DA . The interplay between oxidative stress and fatty acids profile in Romanian spotted cows with placental retention. Vet Sci. (2024) 11:499. doi: 10.3390/vetsci11100499, 39453091 PMC11512347

[153] Rafa H Oroian I Cozma OM Morohoschi AG Dumitraș DA Ștefănuț CL . Peripartal changes of metabolic and hormonal parameters in Romanian spotted cows and their relation with retained fetal membranes. Front Vet Sci. (2024) 11:9666. doi: 10.3389/fvets.2024.1409666, 38846787 PMC11153820

[154] Shimizu T Morino I Kitaoka R Miyamoto A Kawashima C Haneda S . Changes of leukocyte counts and expression of pro- and anti-inflammatory cytokines in peripheral leukocytes in periparturient dairy cows with retained fetal membranes. Anim Sci J. (2018) 89:1371–8. doi: 10.1111/asj.13065, 29956439

[155] Srinivas M Thangamani A Chandra Prasad B Kumar LP. Hemato-biochemical evaluation of Murrah buffaloes affected with retained fetal membranes. Intas Polivet. (2018) 19:189–91.

[156] Verma AK Upadhayay S Bhordia A Singh A Deshdeepak Kumar A. Clinical intervention of puerperal metritis in Indian water buffaloes (*Bubalus bubalis*) in Meerut, Uttar Pradesh. Int J Appl Res Vet Med. (2021) 19:33–8.

[157] Kumari S Kumaresan A Patbandha TK Ravi SK. Risk factors for metritis and its effect on productive and reproductive performance in dairy cattle and buffaloes. Agric Res. (2016) 5:72–80. doi: 10.1007/s40003-015-0183-5

[158] Azawi OI Omran SN Hadad JJ. Treatment of toxic puerperal metritis in Iraqi buffalo cows. Vet Arhiv. (2008) 78:487–99.

[159] Bajaj NK Shukla SP Agrawal RG Agrawal S Honparkhe M. Subclinical endometritis in postpartum buffaloes: an emerging threat. J Anim Res. (2016) 6:819. doi: 10.5958/2277-940X.2016.00104.2

[160] Rahawy M. Study on the post-partum disorders and their relationship with the reproductive performance in Iraqi cow-buffaloes. Iraqi J Vet Sci. (2021) 35:313–7. doi: 10.33899/ijvs.2020.126771.1387

[161] Fagiolo A Lai O. Mastitis in buffalo. Ital J Anim Sci. (2007) 6:200–6. doi: 10.4081/ijas.2007.s2.200

[162] Burvenich C Van Merris V Mehrzad J Diez-Fraile A Duchateau L. Severity of *E. coli* mastitis is mainly determined by cow factors. Vet Res. (2003) 34:521–64. doi: 10.1051/vetres:2003023, 14556694

[163] Abdullah M Javed K Khalid MS Ahmad N Bhatti JA Younas U. Relationship of udder and teat morphology with milk production in Nili-Ravi buffaloes of Pakistan. Buffalo Bull. (2013) 32:1335–8.

[164] Borghese A Rasmussen M Thomas CS. Milking management of dairy buffalo. Ital J Anim Sci. (2007) 6:39–50. doi: 10.4081/ijas.2007.s2.39

[165] Boselli C Mazzi M Borghese A Terzano G Giangolini G Filippetti F . Milk flow curve and teat anatomy in mediterranean Italian buffalo cows. Rev Vet. (2010) 21:573–8.

[166] Hussain R Javed MT Khan A Muhammad G. Risks factors associated with subclinical mastitis in water buffaloes in Pakistan. Trop Anim Health Prod. (2013) 45:1723–9. doi: 10.1007/s11250-013-0421-4, 23712397

[167] Singha S Koop G Ceciliani F Derks M Hoque MA Hossain MK . The prevalence and risk factors of subclinical mastitis in water buffalo (Bubalis bubalis) in Bangladesh. Res Vet Sci. (2023) 158:17–25. doi: 10.1016/j.rvsc.2023.03.004, 36907020

[168] Catozzi C Sanchez Bonastre A Francino O Lecchi C De Carlo E Vecchio D . The microbiota of water buffalo milk during mastitis. PLoS One. (2017) 12:e0184710. doi: 10.1371/journal.pone.0184710, 28926595 PMC5604978

[169] Singha S Ericsson CD Chowdhury S Nath SC Paul OB Hoque MA . Occurrence and aetiology of subclinical mastitis in water buffalo in Bangladesh. J Dairy Res. (2021) 88:314–20. doi: 10.1017/S0022029921000698, 34412714

[170] Salvador RT Beltran JMC Abes NS Gutierrez CA Mingala CN. Short communication: prevalence and risk factors of subclinical mastitis as determined by the California mastitis test in water buffaloes (Bubalis bubalis) in Nueva Ecija, Philippines. J Dairy Sci. (2012) 95:1363–6. doi: 10.3168/jds.2011-4503, 22365218

[171] Kaur M Verma R Bansal BK Mukhopadhyay CS Arora JS. Status of sub-clinical mastitis and associated risk factors in Indian water buffalo in Doaba region of Punjab, India. *Indian*. J Dairy Sci. (2015) 68:2015.

[172] Cavallina R Roncoroni C Campagna MC Minero M Canali E. Buffalo behavioural response to machine milking in early lactation. Ital J Anim Sci. (2008) 7:287–95. doi: 10.4081/ijas.2008.287

[173] De Rosa G Grasso F Braghieri A Bilancione A Di Francia A Napolitano F. Behavior and milk production of buffalo cows as affected by housing system. J Dairy Sci. (2009) 92:907–12. doi: 10.3168/jds.2008-1157, 19233783

[174] Ng L Jost C Robyn M Dhakal IP Bett B Dhakal P . Impact of livestock hygiene education programs on mastitis in smallholder water buffalo (*Bubalus bubalis*) in Chitwan, Nepal. Prev Vet Med. (2010) 96:179–85. doi: 10.1016/j.prevetmed.2010.06.012, 20655119 PMC3001402

[175] Wente N Krömker V. *Streptococcus dysgalactiae*—contagious or environmental? Animals. (2020) 10:2185. doi: 10.3390/ani10112185, 33266438 PMC7700458

[176] Tripaldi C Palocci G Miarelli M Catta M Orlandini S Amatiste S . Effects of mastitis on buffalo milk quality. Asian-Australasian J Anim Sci. (2010) 23:1319–24. doi: 10.5713/ajas.2010.90618

[177] Costa A Neglia G Campanile G De Marchi M. Milk somatic cell count and its relationship with milk yield and quality traits in Italian water buffaloes. J Dairy Sci. (2020) 103:5485–94. doi: 10.3168/jds.2019-18009, 32229124

[178] Wall SK Hernández-Castellano LE Ahmadpour A Bruckmaier RM Wellnitz O. Differential glucocorticoid-induced closure of the blood-milk barrier during lipopolysaccharide- and lipoteichoic acid-induced mastitis in dairy cows. J Dairy Sci. (2016) 99:7544–53. doi: 10.3168/jds.2016-11093, 27372589

[179] Bharti PK Dutt T Patel BHM Pandey HO Tomar AKS. Does age at weaning influences behaviour of Murrah buffalo calves under semi-intensive management conditions? Indian J Anim Sci. (2015) 85:1031–6. doi: 10.56093/ijans.v85i9.51749

[180] Sikka P Sethi RK Tomer AKS Chopra SC. Blood metabolites levels in relation to age and live weight in young buffalo calves. Asian-Australasian J Anim Sci. (1994) 7:201–5. doi: 10.5713/ajas.1994.201

[181] Shao B Sun H Ahmad MJ Ghanem N Abdel-Shafy H Du C . Genetic features of reproductive traits in bovine and Buffalo: lessons from bovine to Buffalo. Front Genet. (2021) 12:617128. doi: 10.3389/FGENE.2021.617128/FULL, 33833774 PMC8021858

[182] Li W Xie Q Zheng H Duan A Huang L Feng C . Genome-wide association study identifies potential regulatory loci and pathways related to Buffalo reproductive traits. Genes. (2025) 16:422. doi: 10.3390/GENES16040422, 40282382 PMC12026540

[183] Zhou J Liu C Feng W Deng T He H Yang H . Genetics and reproduction in buffalo: latest insights into the genetic architecture of reproductive traits. J Reprod Develop. (2026) 72:421–9. doi: 10.1262/JRD.2025-117, 42309754 PMC13286691

[184] Li J Liu J Campanile G Plastow G Zhang C Wang Z . Novel insights into the genetic basis of buffalo reproductive performance. BMC Genomics. (2018) 19:814. doi: 10.1186/S12864-018-5208-6, 30419816 PMC6233259

[185] Vohra V Chhotaray S Gowane G Alex R Mukherjee A Verma A . Genome-wide association studies in Indian Buffalo revealed genomic regions for lactation and fertility. Front Genet. (2021) 12:696109. doi: 10.3389/fgene.2021.696109, 34616425 PMC8488374

[186] Mishra DC Bhati J Yadav S Avashthi H Sikka P Jerome A . Comparative expression analysis of water buffalo (*Bubalus bubalis*) to identify genes associated with economically important traits. Front Vet Sci. (2023) 10:1160486. doi: 10.3389/fvets.2023.1160486, 37252384 PMC10213454

[187] Ciornei SG Drugociu D Roșca P Solcan G. Caesarean section in uterine torsion at buffalo. Rev Rom Med Vet. (2020) 30:53–6.

[188] Ciornei SG Drugociu D Roșca P Solcan G. Ovarian hypofunction in the Carpathian indigenous buffalo. Rev Rom Med Vet. (2021) 31:67–73.

[189] Ciornei SG Roşca P. Upgrading the fixed-time artificial insemination (FTAI) protocol in Romanian buffaloes. Front Vet Sci. (2023) 10:1265060. doi: 10.3389/fvets.2023.1265060, 37964914 PMC10641460

